# Interface Engineering, Charge Carrier Dynamics, and Solar-Driven Applications of Halide Perovskite/2D Material Heterostructured Photocatalysts

**DOI:** 10.1021/acsami.4c20972

**Published:** 2025-04-11

**Authors:** Haihang Tong, Fang-Fang Li, Minshu Du, Haisheng Song, Bin Han, Guohua Jia, Xue-Qing Xu, Xingli Zou, Li Ji, Ji-Jung Kai, Zheng Hu, Hsien-Yi Hsu

**Affiliations:** † School of Energy and Environment, Department of Materials Science and Engineering, Centre for Functional Photonics (CFP), City University of Hong Kong, Kowloon Tong, Hong Kong 999077, China; ‡ School of Materials Science and Engineering, Huazhong University of Science and Technology, 1037 Luoyu Road, Wuhan, Hubei 430074, China; § School of Materials Science and Engineering, Northwestern Polytechnical University, Xi’an, Shaanxi 710072, China; ∥ Wuhan National Laboratory for Optoelectronics (WNLO) and School of Optical and Electronic Information, Huazhong University of Science and Technology, 1037 Luoyu Road, Wuhan, Hubei 430074, P. R. China; ⊥ Materials Institute of Atomic and Molecular Science, Shaanxi University of Science and Technology, Xi’an 710021, China; # Curtin Institute of Functional Molecules and Interfaces, School of Molecular and Life Sciences, Curtin University, GPO Box U1987, Perth, WA 6845, Australia; g Key Laboratory of Renewable Energy, Guangdong Provincial Key Laboratory of New and Renewable Energy Research and Development, Guangzhou Institute of Energy Conversion, Chinese Academy of Sciences, Guangzhou 510640, P.R. China; h State Key Laboratory of Advanced Special Steel & Shanghai Key Laboratory of Advanced Ferrometallurgy & School of Materials Science and Engineering, Shanghai University, Shanghai 200444, China; i State Key Laboratory of ASIC and System, School of Microelectronics, Fudan University, Shanghai 200433, China; j Department of Mechanical Engineering, City University of Hong Kong, Kowloon Tong, Hong Kong 999077, China; k Key Laboratory of Mesoscopic Chemistry of MOE and Jiangsu Provincial Laboratory for Nanotechnology, School of Chemistry and Chemical Engineering, Nanjing University, Nanjing 210023, China; l Shenzhen Research Institute of City University of Hong Kong, Shenzhen 518057, P. R. China

**Keywords:** halide perovskites, 2D materials, photocatalysis, heterostructure, interface, dynamics

## Abstract

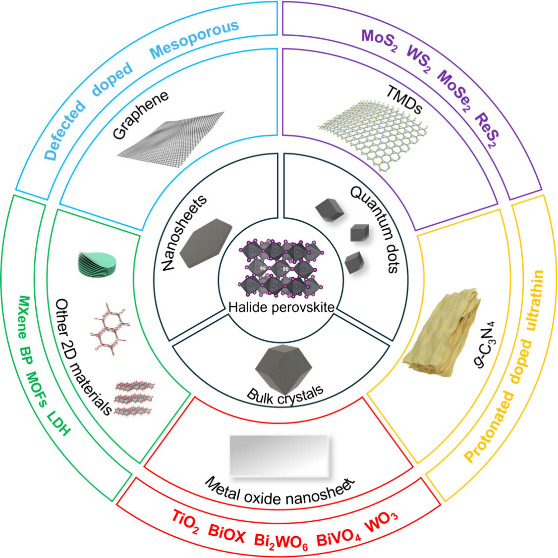

Halide perovskites (HPs), renowned for their intriguing optoelectronic properties, such as robust light absorption coefficient, long charge transfer distance, and tunable band structure, have emerged as a focal point in the field of photocatalysis. However, the photocatalytic performance of HPs is still inhibited by rapid charge recombination, insufficient band potential energy, and limited number of surface active sites. To overcome these limitations, the integration of two-dimensional (2D) materials, characterized by shortened charge transfer pathways and expansive surface areas, into HP/2D heterostructures presents a promising avenue to achieve exceptional interfacial properties, including extensive light absorption, efficient charge separation and transfer, energetic redox capacity, and adjustable surface characteristics. Herein, a comprehensive review delving into fundamentals, interfacial engineering, and charge carrier dynamics of HP/2D material heterostructures is presented. Numerous HP/2D material photocatalysts fabricated through diverse strategies and interfacial architectures are systematically described and categorized. More importantly, the enhanced charge carrier dynamics and surface properties of the HP/2D material heterostructures are thoroughly investigated and discussed. Finally, an analysis of the challenges faced in the development of HP/2D photocatalysts, alongside insightful recommendations for potential strategies to overcome these barriers, is provided.

## 1 Introduction

Utilizing renewable solar energy through photocatalytic processes is one of the most promising ways to address the increasing energy crisis and environmental pollution. As innovative semiconductor materials, halide perovskites (HPs) have witnessed remarkable development and are regarded as new-generation photocatalysts owing to their intriguing photophysical and electrochemical properties, such as strong light absorption coefficient, long charge carrier diffusion distance, tunable band gap, low trap densities, and fast carrier mobility.^1−9^ Since the pioneering work of Park et al. demonstrated photocatalytic hydrogen production using MAPbI_3_ in its saturated solution, HP photocatalysts have led to outstanding achievements in H_2_ evolution, CO_2_ reduction, pollutants degradation, and organic synthesis.^10−16^

Achieving high photocatalytic performance for HPs is a central goal and requires understanding the photocatalysis process. The basic photocatalysis process generally consists of three steps: 1) light absorption by photocatalyst; 2) photogeneration of charge carriers and their separation and migration; and 3) surface redox reactions on the photocatalyst surface. HPs have a strong absorption coefficient and narrow band gap, which gives them a broad solar spectrum absorption range and allows them to produce large amounts of charge carriers upon light irradiation.^17−21^ In addition, the low exciton binding energy and high carrier mobility contribute to HPs’ efficient separation and the fast migration of photogenerated electrons and holes. As a consequence, it is highly possible that the photogenerated charge carriers can migrate to the surfaces of HPs before recombination. Despite these fascinating merits, HPs may still face challenges in their kinetics and thermodynamics. In a typical photocatalysis process, the time scale of surface redox reactions is about 10^–3^–10^–1^ s, which is much higher than those of light absorption (10^–15^–10^–9^ s), charge separation and migration (<10^–15^ s), and even recombination (10^–7^–10^–6^ s),^22−29^ indicating severe charge carrier recombination. Moreover, an ideal photocatalyst is expected to possess broad light absorption and robust redox activity, but the negative conduction band (CB) and narrow band gap of most HPs mean that many oxidation reactions cannot occur on their high valence band (VB). Therefore, HPs alone may not be able to realize intricate redox reactions requiring high energy and fast kinetics, and a modification strategy is necessary.

**Figure 1:**
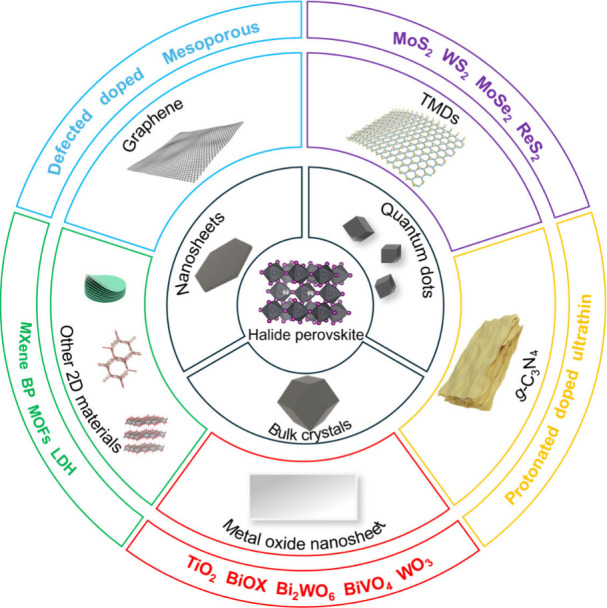
Halide perovskite and 2D materials.

Coupling HPs with another material that acts as a photocatalyst or cocatalyst to form a heterostructure is a straightforward approach to effectively promote the separation of photogenerated charge carriers. Notably, the second material can significantly enhance the surface reaction activity through facilitating the transfer of electrons or holes between the photocatalyst surface and the reaction medium as well as providing large amounts of surface active sites. In the pursuit of higher photocatalytic activity of HP-based heterostructure photocatalysts, the search for the second semiconductor/cocatalyst has unveiled a wide range of metal-, oxide-, carbon-, nitride-, and chalcogenide-based materials. Besides composition, the dimensional shape of the material also plays a significant role in the construction of the heterostructure. Two-dimensional (2D) materials with unique physical and chemical characteristics are gaining tremendous interest and becoming the preferred choice to combine with HPs in many studies. Since 2017, reports on HP/2D material heterostructure photocatalysts have consecutively emerged. These 2D materials include but are not limited to metal oxides, graphitic carbon nitride (g-C_3_N_4_), graphene, transition metal dichalcogenides (TMDs), black phosphorus (BP), MXenes, and metal–organic frameworks (MOFs) (Figure 1). Compared to their 3D bulk counterparts, the vast surface area of 2D materials not only excels at harvesting light but also provides a large contact area with abundant binding sites for strong interactions with other photocatalysts, along with the creation of sufficient charge trapping and transfer channels. Additionally, their thin nature shortens the migration path of charge carriers, accompanied by suppressed electron–hole recombination. Moreover, a plethora of unsaturated atoms exposed on the surface can serve as active sites and accelerate the molecular adsorption and redox reactions.^30^ Hence, an optimum HP/2D material heterostructure with broad light absorption, efficient charge carrier separation, and high surface reactivity can be obtained. In particular, more insightful comprehension of the interfacial charge carrier dynamics will be conducive to leveraging the potential of both HPs and 2D materials, as the mechanisms of charge carrier behavior at the interfaces between HPs and 2D materials, as well as photocatalysts and the reaction medium, are significant for photocatalytic performance.

Despite the substantial growth of research on HP/2D materiald heterostructure photocatalysts with unexpected performances in solar-driven photocatalysis applications, very few articles systematically summarize these developments and provide comprehensive discussion. This Review introduces HP/2D materials heterostructure photocatalysts commencing with the fundamentals of HPs and 2D materials and focusing on the interface-associated interactions and charge carrier dynamics, followed by elaboration of the enhanced surface properties of HP/2D materials photocatalysts for various of applications. Finally, ongoing challenges and future perspectives are proposed. Our Review aims to provide an insightful understanding of the interfacial characteristics of HP/2D materials, with the goal of sparking novel ideas for the design of highly efficient HP/2D materials heterostructure photocatalysts with leveraged synergistic properties.

## 2 Fundamentals of Halide Perovskites and 2D Materials

### 2.1 Structural and Optoelectronic Properties

**Figure 2:**
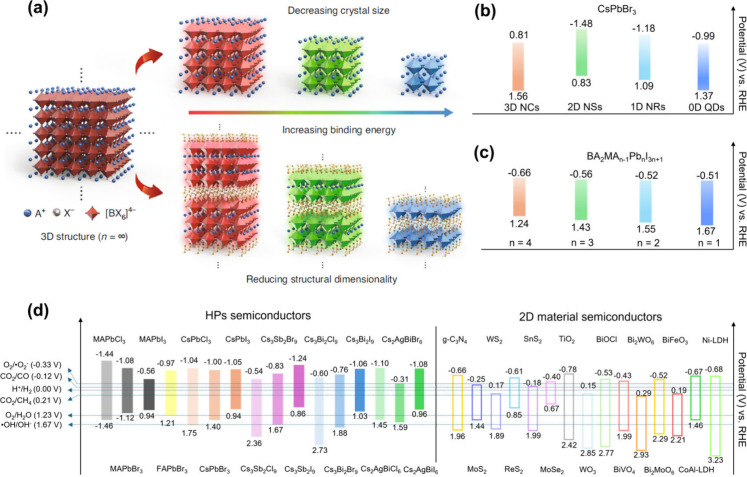
(a) Schematic illustration of a perovskite with decreased size and reduced dimension. Reproduced with permission from ref (50). Copyright 2021, Springer Nature. (b) Band structure of CsPbBr_3_ with different morphology sizes. Data collected from refs (51−54). (c) Band structure of BA_2_MA_*n*-1_Pb_*n*_I_3*n*+1_ HPs. Data collected from ref (36). (d) Band structure of HPs (MAPbX_3_ (X = Cl, Br, I),^55−57^ FAPbBr_3_,^58^ CsPbX_3_ (X = Cl, Br, I),^59−61^ Cs_3_Sb_2_X_3_ (X = Cl, Br, I),^62,63^ Cs_3_Bi_2_X_9_ (X = Cl, Br, I),^64^ Cs_2_AgBiX_6_ (X = Cl, Br, I),^65−67^ and 2D material semiconductors (g-C_3_N_4_,^68^ MoS_2_,^69^ WS_2_,^70^ ReS_2_,^71^ SnS_2_,^72^ MoSe_2_,^73^ TiO_2_,^74^ WO_3_,^75^ BiOCl,^76^ BiVO_4_,^52^ Bi_2_WO_6_,^77^ Bi_2_MoO_6_,^78^ BiFeO_3_,^79^ CoAl-LDH,^80^ and Ni-MOF^53^) coupled with HPs.

In general, HPs exhibit the formula ABX_3_, where A can be a monovalent organic cation (e.g., CH_3_NH_3_^+^ (MA^+^) or CH(NH_2_)_2_^+^ (FA^+^)) or an inorganic cation (e.g., Cs^+^), B is a divalent metal cation (e.g., Pb^2+^ or Sn^2+^), and X is a halide anion (e.g., Cl^–^, Br^–^, or I^–^).^31−34^ The composition and structure tunability largely expand the possibilities for HPs at the levels of both structure configuration and morphology.^35^ Interestingly, the transformation of dimension could influence the band structure of HPs. As shown in Figure 2a, with the reduction of size in terms of morphology, HPs change from 3D bulk to 2D sheets and 0D quantum dots (QDs). Taking CsPbBr_3_ as an example, its corresponding 3D nanocrystals (NCs), 2D nanosheets (NSs), 1D nanorods (NRs), and 0D QDs show different band structures (Figure 2b). This property also offers different coupling modes for HPs with 2D materials in photocatalysis. Meanwhile, for the lattice structure, the regulation of the A site through employing large organic groups can lead to the formation of quasi-2D HPs presenting the formula of (A′)_*m*_(A)_*n*-1_B_*n*_X_3*n*+1_. Recently, quasi-2D HPs have emerged as photocatalysts for H_2_ evolution. Fu et al. used CH_3_(CH_2_)_3_^+^ (BA^+^) as a large intercalation organic cation to construct 2D/quasi-2D BA_2_MA_*n*-1_Pb_*n*_3_*n*+1_ HPs. As the value of *n* increases from 1 to 4, the band gap and band edge (the conduction band (CB) and valence band (VB)) of BA_2_MA_*n*-1_Pb_*n*_I_3*n*+1_ HPs show a gradual decrease and negative shift, respectively (Figure 2c).^36^ In addition, the adjustable composition contributes to HPs’ abundant optoelectronic structures (Figure 2d). Compared to the A site, the variations of the B site and halogen species have a more significant influence on both the band gap and band positions, which may be ascribed to the dominant contribution of the BX_6_ octahedron. With the X site variation from Cl to I, the band gap of HPs presents a decreasing trend, while the narrower band gap with a broader light absorption range simultaneously indicates a deficient redox capability. Though the bromine and iodine HP derivatives generally have excellent reduction activity due to their relatively high CBs and narrow band gaps, the negative VBs inhibit some energetically demanding oxidation reactions.

2D materials represent a diverse class of materials distinguished by their unique atomic-scale thickness coupled with extensive lateral dimensions. Beyond conventional nanosheets, the introduction of emerging architectures, such as ultrathin nanobelts and nanoplates, has significantly broadened the scope of 2D materials. These materials are predominantly classified as van der Waals layered structures, encompassing graphene oxide (GO), TMDs, BP, and g-C_3_N_4_.^37^ Furthermore, the category of 2D materials also includes metal oxides, which can be subdivided into layered metal oxides such as bismuth-based oxides, perovskite layered oxides, and layered double hydroxides (LDHs), as well as ultrathin nonlayered metal oxides, exemplified by TiO_2_, WO_3_, and Fe_2_O_3_.^38−40^ Notably, certain ultrathin MOFs and covalent organic frameworks (COFs) also belong to the class of 2D materials.^41^ In contrast to HPs, many 2D material semiconductors like g-C_3_N_4_ and metal oxides. the VBs of which show robust oxidation potential, are ideal candidates for coupling with HPs and can effectively compensate for the deficiencies of HPs (Figure 2d). Despite the narrow band gap of TMDs and other layered 2D materials, such as graphene, MXene, and BP, their superior conductivity can significantly accelerate the transfer of photoexcited charge carriers.

The manipulation of dimension can also alter the optoelectronic properties of 2D materials. The relationship between the change (Δ*E*_g_) in energy band gap (*E*_g_) and the thickness (*L*_*z*_) of 2D materials can be expressed by eq 1,^42^
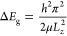
1where μ is the reduced effective mass of the exciton. With the decrease of dimension or thickness, the band gap shows enlargement accompanied by the band edge positions separating in opposite directions. Therefore, controlling the dimensionality of semiconductors allows for modification in their electronic structure, providing a versatile tool to fine-tune the system’s performance. Studies have shown that g-C_3_N_4_ sheets which vary in thickness achieved through exfoliation demonstrate diverse photocatalytic performances in CO_2_ reduction. With the thickness decreasing from 93 nm of bulk to 0.8 nm of nanosheets, the CB of g-C_3_N_4_ shifts to a negative position, which endows higher reduction potential favoring the reduction of CO_2_. Besides, reduced thickness also leads to remarkable exposure of active sites as well as inhibition of electron–hole pairs.^43^ For another class of 2D materials, the electronic band structures of TMDs are intimately associated with their thickness or layer numbers. Synthesized TMDs (MoS_2_, MoSe_2_, WS_2_, WSe_2_) present an extended band gap with the layer (L) numbers varying from 3L to 1L due to the quantum confinement effect, which is in accordance with the theoretical calculation results.^44^ The tunability of the electronic band structure via layer adjustment of TMDs gives them flexible activity in photocatalysis applications. It is reported that a series of WO_3_/Pt/WS_2_ photocatalysts show different photocatalysis performance on pollutants degradation and H_2_ evolution, and the best performance was achieved by one with 2–3L WS_2_ owing to its suitable band structure and ultrahigh electron conductivity and mobility.^45^ Another feature of 2D materials originating from the confinement is the transition between a direct band gap and an indirect band gap. For example, when the thickness of MoS_2_ decreases to monolayer, it changes from an indirect to a direct band gap semiconductor. A similar phenomenon is also observed in Cs_2_AgBiBr_6_, which undergoes an indirect-to-direct band gap transition when its thickness is reduced to monolayer, along with the increase of band gap.^46^ Moreover, the carrier mobility and exciton binding energy of Cs_2_AgBiBr_6_ are tunable with variation of thickness, providing the potential for design of desirable photocatalysts. Essentially, the quantum confinement effects of 2D materials regulate electron–hole pairs, forming bound excitons with significantly lower binding energy compared to their bulk equivalents. This contributes to enhanced electronic properties and charge carrier dynamics at material surfaces.

In contrast to bulk materials, where excited carrier recombination typically occurs midway, limiting the number of carriers reaching the surface and thereby reducing the photocatalytic efficiency, the transport distance in 2D materials is significantly shortened. This reduction facilitates a faster migration of excited carriers. The charge diffusion time, *t*, can be calculated according to the diffusion equation (eq 2),
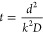
2where *d* is the particle size, *k* is the diffusion constant, and *D* is the diffusion coefficient. Obviously, the parameter *d*, which represents the layer thickness, has a direct proportionality to the diffusion time.^47^

The unique ionic nature of HPs makes them highly tolerant to bulk defects and deep trap states, which contributes to longer carrier diffusion length and charge carrier lifetime compared to traditional semiconductors.^48^ This means that there is a higher possibility for photoinduced charges to reach the surface of HPs and participate in redox reactions during the photocatalysis process. For 2D materials, surface defects can be easily created and play a significant role in photocatalysis. Surface defects can regulate the electronic structure and act as traps for photogenerated electrons and holes to suppress their recombination. Additionally, surface defects could induce unsaturated coordination configurations, thus making the surface more active for adsorption and reaction.^49^ The minimal thickness and large surface area of the 2D materials provide a desirable platform for creating surface defects. Representative examples such as C or N vacancy in carbon nitrides, oxygen vacancy in metal oxides, and sulfur vacancy in metal sulfides have demonstrated their superior enhancement of catalysis applications.^49^ Different from HPs, which have long charge diffusion lengths, 2D materials generally exhibit shortened charge diffusion lengths due to the fast charge transfer to the surface in a short time, which facilitates the photocatalysis process.

Consequently, the distinctive properties of 2D materials confer significant advantages over their 3D bulk counterparts, making them particularly attractive for photocatalytic applications. Key attributes include the following: (i) a tunable band structure, allowing precise adjustment of the band gap to optimize absorption in the visible light spectrum and enhance light-harvesting efficiency under solar illumination; (ii) an exceptionally high specific surface area, which increases the availability of active sites for catalytic reactions; (iii) minimized recombination of charge carriers, as the ultrathin nature of 2D materials shortens the transport distances for excited electron–hole pairs, facilitating more efficient charge separation and transfer; and (iv) superior mechanical strength and enhanced electrical conductivity, which improve the durability and overall performance of the photocatalyst. Together, these unique features enable 2D materials to exhibit outstanding photocatalytic activity, positioning them as ideal candidates for integration with HPs to achieve enhanced performance in solar-driven applications.

### 2.2 Role of 2D Materials in HP/2D Material Heterostructures

#### 2.2.1 Serving as a Robust Substrate

**Figure 3:**
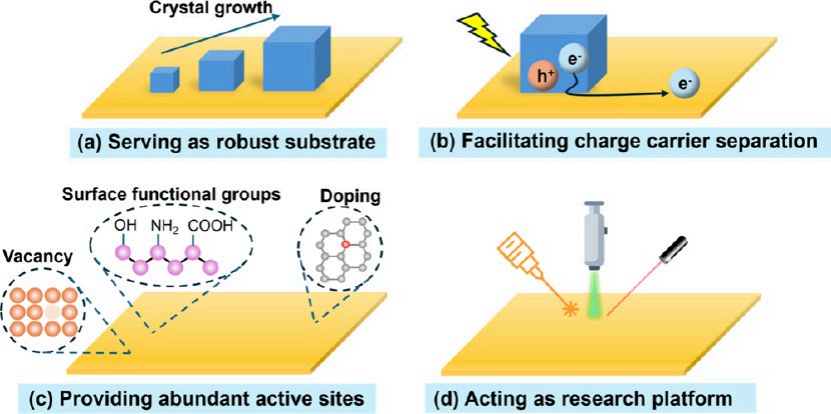
Roles of 2D materials in coupling with halide perovskites.

As illustrated in Figure 3a, 2D materials are ideal platforms for the loading of HPs. On the one hand, the exceptionally large surface area enables uniform dispersion of HPs on the surface of 2D materials. On the other hand, the abundant surface functional groups offer large amounts of sites for the nucleation of HPs and facilitate in situ growth. These synergistic features foster strengthened physical and chemical interactions between HPs and 2D materials. Such robust interactions not only are conducive for subsequent interfacial dynamic processes but also improve stability, which is particularly beneficial in harsh environments where maintaining the integrity of the composite structure is essential for long-term performance. Moreover, the extensive surface area of 2D material semiconductors enables them to fully exploit their optical performance.^81^ The good dispersion of HPs effectively avoids their agglomeration and, hence, increases the light-harvesting area.

#### 2.2.2 Promoting Charge Separation and Transfer

The severe recombination of photogenerated electrons and holes limits the photocatalytic activity of HPs. Incorporating 2D materials with HPs to form a heterostructure is one of the most effective approaches to solve this problem (Figure 3b). 2D materials with excellent conductivity or metal characteristics such as graphene, MXenes, and TMDs are desirable cocatalysts for HPs. Their lower Fermi levels relative to the CB of HPs ensure the migration of photoexcited electrons from HPs to them, thereby promoting charge separation and transfer. Moreover, their high conductivity further accelerates electron transfer. HPs can also combine with 2D material semiconductors such as g-C_3_N_4_ and metal oxides. The discrepancy between the band structures (e.g., band positions and Fermi levels) could lead to the formation of energy-staggered heterostructures with a built-in electric field (BIEF) at the interface. Upon light irradiation, this powerful BIEF could drive the separation and transfer of photoinduced charges through different pathways, ultimately enhancing the photocatalysis performance. It is noteworthy that, compared to the 3D bulk materials, the ultrathin property of 2D materials can significantly shorten the charge diffusion distance and facilitate charge transfer.^47^

#### 2.2.3 Providing Abundant Surface Active Sites

Surface reactivity is also a dominant factor in photocatalysis reactions. 2D materials, characterized by their extensive diversity, offer numerous surface types that promote surface reactions. Combined with their large surface area, abundant surface active sites on 2D materials can greatly help the catalytic reactions to proceed by enhancing the adsorption and selectivity of target reactants (Figure 3c). Notably, the surface functionality of 2D materials can be further optimized via various modification strategies such as elemental doping, defects creation, morphology regulation, and so on. The highly adjustable surface of 2D materials enables HP/2D material heterostructures to have exceptional catalytic performance across various reactions including CO_2_ reduction, H_2_ evolution, organic conversion, and pollutant degradation. One noticeable characteristic of 2D materials is that the edge sites with unsaturated coordination exhibit distinct activities. The well-exposed edge sites allow 2D materials to facilitate unique reactivity compared to common bulk materials, leading to higher efficiency and selectivity in the photocatalytic process.

#### 2.2.4 Acting as an Ideal Platform for Mechanism Investigation

Unlike their 3D bulk counterparts, 2D materials with atomic-scale thickness could be employed as ideal platforms to investigate photocatalytic reaction processes (Figure 3d). This is primarily due to the fact that subtle changes can significantly alter the intrinsic optoelectronic characteristics and surface properties of 2D materials, and these changes can be readily reflected by characterizations and experiments. However, for 3D bulk materials, though the catalytic activity could be promoted through modifications, deciphering the mechanisms remains ambiguous since intrinsic features buried within the bulk are less susceptible to external influences. Therefore, integrating HPs with 2D materials will hopefully construct a typical form for studying the photocatalytic mechanisms in HP-based heterostructures. Such HP/2D material heterostructures are expected to provide valuable insights into both charge carrier dynamics and surface reactions. The synergistic combination of HPs and 2D materials could enable more precise control and observation of photocatalytic processes, thereby advancing our understanding and facilitating the development of more efficient photocatalytic systems.

### 2.3 Fundamentals of Heterostructures

**Figure 4:**
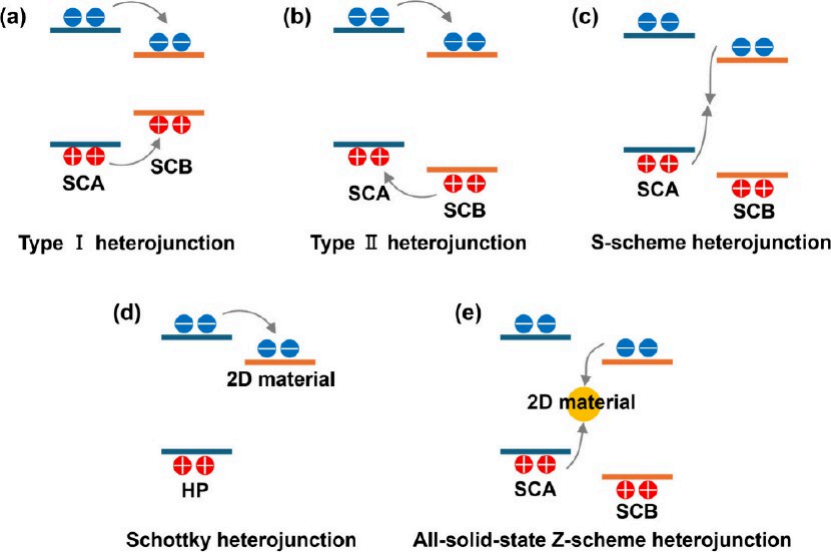
Schematic illustration of heterojunction types: (a) type I heterojunction; (b) type II heterojunction; (c) S-scheme heterojunction; (d) Schottky heterojunction; and (e) all-solid-state heterojunction.

The extensive diversity and tunability of both HPs and 2D materials make them well-suited for forming heterostructures with each other. Different energy bands between the two materials result in various charge transfer pathways at the interfaces. Based on the band structures and charge carrier transfer mechanisms, HP/2D material heterostructures can be typically classified into several types, including type I, type II, Schottky junction, S-scheme heterojunction, and Z-scheme heterojunction (Figure 4).

Type I heterojunctions form when the conduction band (CB) and valence band (VB) of semiconductor B (SCB) are encompassed by those of semiconductor A (SCA) (Figure 4a). Typically, upon light irradiation, photoinduced electrons and holes from SCA (MAPbI_3_) migrate, respectively, to the CB and VB of SCB (black phosphorus). Charge carriers in SCA can be effectively separated and transferred in type I heterojunctions.^82^ However, these excited electrons and holes undergo energy loss, and their accumulation on the energy bands will suppress the charge separation in SCB, which would lead to unsatisfactory photocatalytic activity. Hence, type I HP/2D material heterojunctions are rarely adopted in photocatalysis reactions that require high energy.

When SCA possesses a higher CB and VB than SCB, a type II heterojunction with staggered band alignment is formed (Figure 4b). In this structure, photogenerated electrons from the CB of SCA transfer to the CB of SCB, while photogenerated holes in the VB of the semiconductor will simultaneously migrate to the VB of SCA. Compared to type I heterojunctions, photoexcited charge carriers are efficiently separated and transferred in both semiconductors. However, type II heterojunctions still suffer from undesirable energy loss, which brings about reduced redox reactivity.^83^ This is attributed to the fact that the CB of SCB and the VB of SCA, which respectively accommodate photogenerated electrons and holes, exhibit weaker reduction and oxidation potentials. It should also be pointed out that excessive electrons (holes) transferred from SCB (A) would induce strong electrostatic repulsion with photogenerated electrons (holes) of SCA (B), thereby inhibiting charge separation and transfer efficiency. To this end, an S-scheme with a similar band alignment but a more effective charge transfer pathway is proposed. In addition to higher CB and VB, SCA should have a Fermi level (lower work function) higher than that of SCB. A BIEF directed from SCA to SCB at the interface will drive the photoelectrons in the CB of SCB to combine with the holes in the VB of SCA. As a result, electrons and holes with high energy are left in the higher CB of SCA and lower VB of SCB (Figure 4c).^30^ Both type II and S-scheme heterojunctions can be obtained through coupling HPs with 2D material semiconductors, and numerous combinations such as HP/metal oxide, HP/g-C_3_N_4_, and HP/TMDs have demonstrated excellent photocatalysis performance.

A Schottky junction can form between HPs and 2D materials with metallic properties (Figure 4d). Representative examples are HP/graphene and HP/MXene heterostructures. The lower Fermi level and extreme electric and thermal conductivities of these 2D materials facilitate swift photoelectron extraction from HPs upon illumination. The equilibrium of Fermi levels between HP and 2D materials leads to a charge space region with the HP’s energy band upward, forming the Schottky barrier. Continuous light irradiation would drive the accumulated electrons on the CB of the HP to transfer to the 2D material, while the Schottky barrier can prevent the electrons’ migration back to the HP, thus ensuring immediate reduction reactions on the surface of the 2D material.^84^ However, the Schottky junction still faces the problem of loss of energy potential. Moreover, these intriguing 2D materials can serve as cocatalysts between two semiconductors to establish an all-solid-state Z-scheme heterojunction (Figure 4f). Acting as a mediator, photoexcited electrons from the CB of SCB would recombine with holes in the VB of SCA with the aid of 2D materials.^85^ Thus, electrons and holes holding high energy potential could be retained for reactions, and high photocatalytic performance can be expected.

## 3 Construction of HP/2D Material Interfaces

### 3.1 Synthesis Strategies

Drawing on the principles of crystal growth and interface interactions, the synthesis strategies of HP/2D material heterostructure photocatalysts can be broadly divided into in situ growth and ex situ combination.

#### 3.1.1 In Situ Growth

In situ growth refers to the formation of a second material on another presynthesized one, a prevalent method for developing HP/2D material heterostructures. In a typical in situ growth process, 2D materials functioning as the substrate are dispersed in the precursors of HPs. Subsequently, small HP crystals nucleate and grow on the surface of the 2D material, resulting in intimate interfaces.^86^ In situ construction of the HP/2D material interface can be achieved through various methods. During the hydrothermal/solvothermal process, 2D materials and the HP precursors (e.g., CsBr and PbBr_2_) are added into an organic solvent with continuous stirring, followed by reaction at high temperature in an autoclave or a three-neck bottle. The hot-injection method involves complicated operations, including the introduction of an inert gas and control of reaction temperature and time, but it could produce HPs with desirable purity and uniform size and shape due to the precise control of the reaction conditions. The antisolvent method is more convenient since it does not require a high reaction temperature; however, the crystallinity of the obtained HPs may be lower than that of HPs prepared under high-temperature conditions. Another facile method of in situ growth is conducted in a saturated solution of HPs. Specifically, the 2D material and as-prepared HP (or its precursors) are first added to the HP’s saturated solution. Then the mixture is heated to dissolve the HP. HP crystals will reprecipitate during the cooling procedure and finally integrate with the 2D material to form heterostructures. The resulting saturated solution containing the HP/2D material could be directly used for photocatalytic H_2_ evolution. Notably, the sizes of HP crystals that grow in situ on 2D materials are generally found to be smaller than those of pure HP crystals. This could be ascribed to the functional groups of 2D materials which facilitate the nucleation process and lead to the formation of more HP nanocrystals.

#### 3.1.2 Ex Situ Combination

Ex situ hybridization is a facile strategy to build HP/2D material heterostructures by directly mixing pre-existent HP and 2D materials. Electrostatic self-assembly is a representative ex situ preparation approach. A crucial factor of this method is that the HP and 2D materials should possess opposite surface charges when dispersed in a liquid medium so that they can bond to each other driven by Coulombic attractive forces with stirring or ultrasonic mixing, leading to the spontaneous formation of stable complexes.^30,87^ This process is finely tunable, allowing for precise control over the spatial arrangement and distribution of the components within the composite. Importantly, the advantage of proceeding under mild conditions enables wide application of this method and amplifies the prospects for large-scale manufacture of HP/2D material heterostructures. Zeta potential analysis is usually performed to examine the surface potential of HP and 2D materials to evaluate the potential of their combination through electrostatic self-assembly. When the zeta potentials of the HP and 2D materials are significantly different, it indicates a strong electrostatic interaction, facilitating the self-assembly process. The larger the magnitude of the zeta potential, the stronger the repulsive or attractive forces between particles, which can enhance the control over the assembly process and the quality of the resulting heterostructures. In addition to the zeta potential, the isoelectric point (IEP) is another important factor that influences the electrostatic interactions between components. It refers to the pH at which a material’s net surface charge is zero, meaning the material has no electrostatic repulsion or attraction.^88^ For effective electrostatic self-assembly, it is crucial that the HP and 2D materials have opposite surface charges at the assembly pH. By adjusting the pH of the dispersion to be above or below the IEP of the materials, one can manipulate the surface charge to optimize the electrostatic attraction between components, which is particularly useful for fine-tuning the self-assembly process and achieving the desired heterostructure morphology. Mechanical hybridization can also realize the combination of HP and 2D materials. The high mechanical energy introduced by ball milling or grinding could induce strong interactions between HP and 2D materials, ensuring their robust integration with each other. However, it is difficult to control the morphology and interface arrangement during this process.

### 3.2 Architectures of HP/2D Material Interfaces

**Figure 5:**
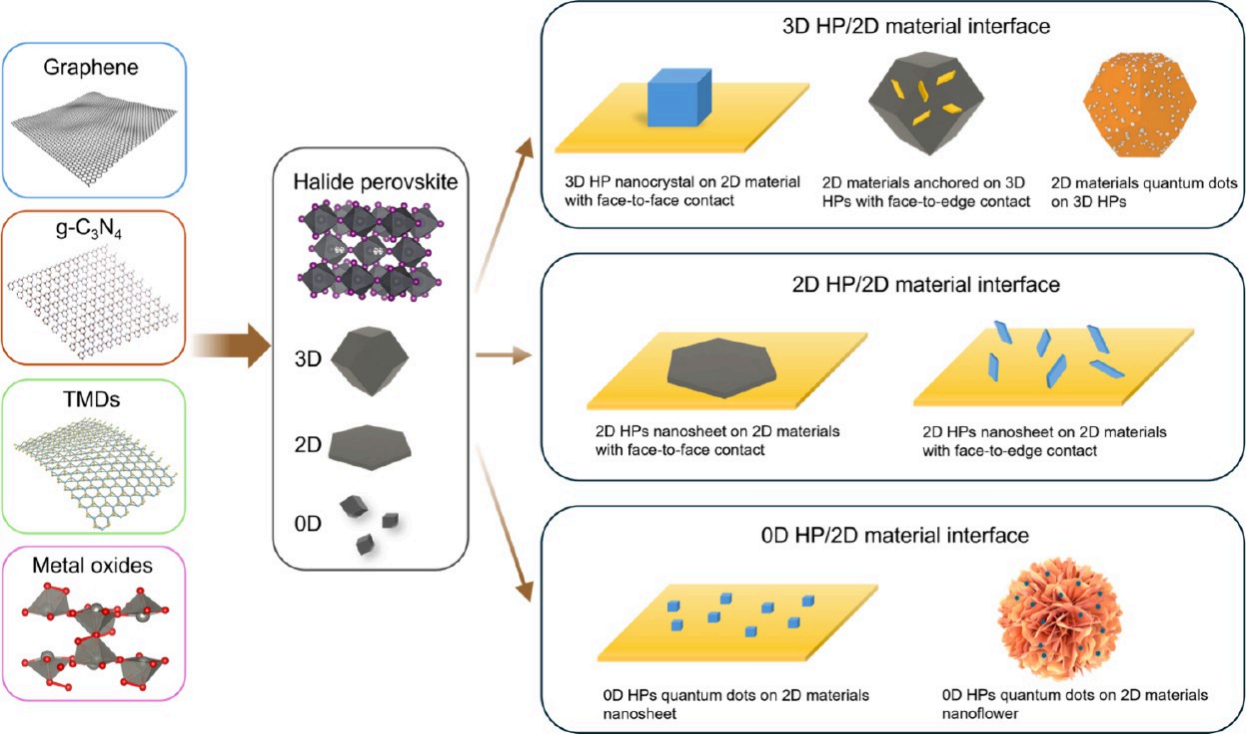
Construction of multiple HP/2D material interfaces.

Crafting interfaces between HP and 2D materials plays a pivotal role in determining both interfacial properties and photocatalyst efficacy, thus emphasizing the importance of developing HP/2D composites. Beyond achieving a well-matched band structure between HP and 2D materials, which facilitates robust light harvesting and efficient charge carrier separation, it is imperative to consider the interfacial contact and the heterointerface mode between them. Various HP/2D material interfaces can be categorized into mainly three types according to dimension: 3D HP/2D material, 2D HP/2D material, and 0D HP/2D material interfaces (Figure 5). This dimensional categorization not only enhances our understanding of the interactions of the materials but also guides the optimization of synthesis protocols to engineer interfaces with tailored properties. Such a strategic interface design is significant for maximizing the photocatalytic performance of HP/2D composites, promising significant advancements in energy conversion and environmental remediation technologies.

#### 3.2.1 3D HP/2D Material Interfaces

**Figure 6:**
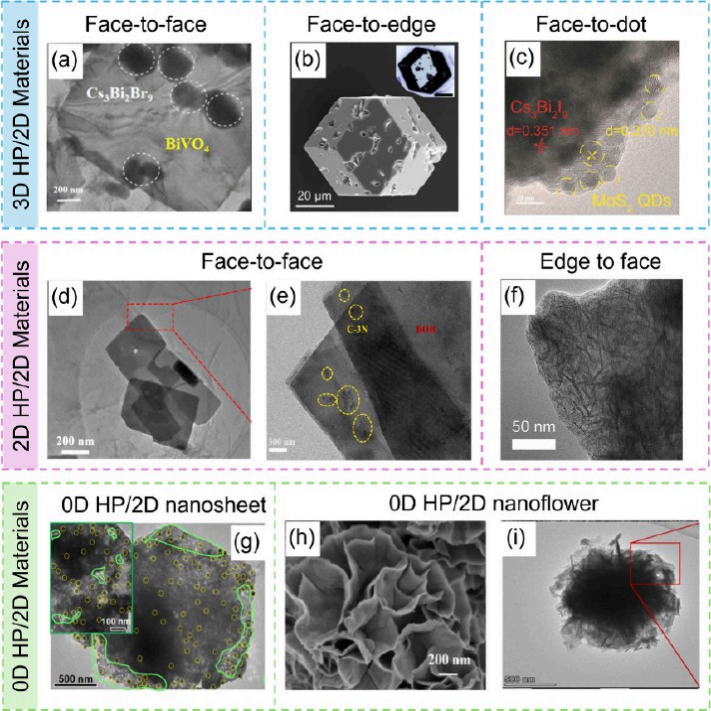
Architectures of the HP/2D material heterostructure. (a) Cs_3_Bi_2_Br_9_/BiVO_4_. Reproduced with permission from ref (89). Copyright 2024, the Royal Society of Chemistry. (b) MoS_2_/MAPbI_3_. Reproduced with permission from ref (69). Copyright 2020, Cell Press. (c) Cs_3_Bi_2_I_9_/MoS_2_ QDs. Reproduced with permission from ref (91). Copyright 2024, the Royal Society of Chemistry. (d) CsPbBr_3_/g-C_3_N_4_. Reproduced with permission from ref (92). Copyright 2022, the Royal Society of Chemistry. (e) CsPbBr_3_/Bi_3_O_4_Br. Reproduced with permission from ref (93). Copyright 2022, Elsevier. (f) Co_*x*_Bi_2–x_O_2_CO_3_/Cs_3_Bi_2_Br_9_. Reproduced with permission from ref (96). Copyright 2023, Elsevier. (g) CsPbBr_3_/Co_3_O_4_. Reproduced with permission from ref (97). Copyright 2023, the Royal Society of Chemistry. (h) Cs_2_SnI_6_/SnS_2_. Reproduced with permission from ref (72). Copyright 2019, American Chemical Society. (i) In_4_SnS_8_/Cs_3_Bi_2_Br_9_. Reproduced with permission from ref (98). Copyright 2022, Elsevier.

3D HP/2D material interfaces are a prevailing strategy for constructing photocatalyst heterostructures with intriguing photocatalytic properties because of the multiple contact modes between the HP and 2D materials. The large surface area of 2D materials provides abundant loading sites for the in situ growth of HP nanocrystals, and a face-to-face interface will be formed. This strategy usually involves HP precursors and as-prepared 2D materials undergoing an antisolvent or heat treatment process. For example, employing BiVO_4_ nanosheets as the substrate and CsBr/BiBr_3_/DMSO as the precursor, Gao et al.^89^ successfully grew Cs_3_Bi_2_Br_9_ particles on BiVO_4_ nanosheets through an antisolvent-assisted in situ assembly method (Figure 6a). The ultrathin BiVO_4_ nanosheets with smooth and large surfaces facilitate the intimate contact and charge transfer between BiVO_4_ and Cs_3_Bi_2_Br_9_, leading to a Cs_3_Bi_2_Br_9_/BiVO_4_ heterostructure with a remarkable Sudan III degradation rate of 99.2% within 40 min of light illumination, which is 2.37 and 106 times higher than those of Cs_3_Bi_2_Br_9_ and BiVO_4_, respectively. Similarly, Zhao et al.^90^ utilized a hot injection method to decorate CsPbBr_3_ (CPB) nanocrystals on the surface of S-doped g-C_3_N_4_ nanosheets (USCN). The successful in situ growth of CsPbBr_3_ on g-C_3_N_4_ and the face-to-face structure were revealed by TEM and AFM. Furthermore, in situ XPS spectroscopy demonstrated the built-in electric field formed at the interface of the S-scheme CPB/USCN heterostructure, which drives the recombination and separation of electrons and holes and endows CPB/USCN an enhanced CO_2_ photoreduction rate of 83.6 μmol g^–1^ h^–1^.

HPs can also serve as the substrate for 2D materials to form the face-to-edge contact mode of 3D HP/2D material interfaces. A typical example is the monolayer MoS_2_ nanosheets/MAPbI_3_ microcrystals (ML-MoS_2_/MAPbI_3_-MCs) composite reported by Zhao et al.^69^ (Figure 6b). Through a recrystallization process dispersing MoS_2_ nanosheets in the saturated solution of MAPbI_3_, MoS_2_ nanosheets were vertically anchored on the surface of MAPbI_3_ microcrystals. Possessing the advantages of light-harvesting MAPbI_3_ and electrocatalytic active MoS_2_, the formed type II ML-MoS_2_/MAPbI_3_-MCs heterostructure exhibited a superior H_2_ evolution rate of 13.6 mmol g^–1^ h^–1^ and solar to hydrogen (STH) efficiency of 1.09%. Ex situ combination is another common strategy to construct 3D HP/2D material interfaces, favored for simplicity and manageability. This strategy is likely to result in a face-to-edge contact mode in which 2D materials are anchored on the surface of 3D HPs. For example, a MAPbBr_3_/La_2_Ti_2_O_7_ heterostructure was synthesized through a straightforward ball milling method. La_2_Ti_2_O_7_ nanosheets were well distributed and inserted on the surface of MAPbBr_3_ blocks. The similar crystal lattice spacing of the (200) plane of MAPbBr_3_ (0.29 nm) and the (002) plane La_2_Ti_2_O_7_ (0.28 nm) facilitates an intimate heterostructure interface between them, and the formation of a type II heterojunction with satisfactory charge carrier separation efficiency givess MAPbBr_3_/La_2_Ti_2_O_7_ a significantly higher photocatalytic CO production rate of 18.9 μmol g^–1^, compared to that of MAPbBr_3_ alone (4.4 μmol g^–1^).^56^ In a recent study, MoS_2_ quantum dots (QDs) were decorated on the surface of Cs_3_Bi_2_I_9_ microcrystals via an electrostatic self-assembly method (Figure 6c). The resulting MoS_2_ QDs/Cs_3_Bi_2_I_9_ showed enhanced charge separation efficiency, and the electron diffusion effect of MoS_2_ QDs accelerated the reduction of protons on the active sites, thereby leading to satisfactory H_2_ evolution performance.^91^

#### 3.2.2 2D HP/2D Material Interfaces

2D HP/2D material interfaces can be divided into two main types: face-to-face and face-to-edge. The face-to-face contact mode ensures a large and intimate heterointerface between the 2D HP and 2D materials, boosting effective charge transfer and separation. Electrostatic self-assembly is a convenient and mild method to construct face-to-face 2D HP/2D material interfaces. This strategy utilizes the difference between the surface potentials of the HP and 2D materials to assemble a stable composite, which is suitable for many 2D materials. For instance, a CsPbBr_3_ nanosheet/g-C_3_N_4_ nanosheet (CPBN/CNN) heterostructure was prepared by immersing CNN in an isopropanol solution containing CPBN and stirring for 2 h (Figure 6d).^92^ A similar process was also adopted in the fabrication of a CsPbBr_3_ nanosheet/Bi_3_O_4_Br nanosheet (CPB/BOB) heterostructure through ultrasonication treatment. With the surface of BOB uniformly covered by CPB, this face-to-face structure could not only promote the charge separation but also supply plentiful active sites for CO_2_ reduction (Figure 6e).^93^ Moreover, it was also reported that Cs_2_AgBiBr_6_ nanosheets (CABB) and Ni-MOF nanosheets were mixed in ethyl acetate with continuous stirring to form a CABB/Ni-MOF composite. The well-aligned energy bands and face-to-face interaction between the two materials guarantee the formation of an S-scheme heterojunction with robust redox potential, resulting in excellent CO_2_ photocatalytic reduction performance.^94^ In addition to self-assembly, the face-to-face mode of 2D HP/2D material interfaces can also be obtained through an in situ growth method. Yue et al.^95^ reported regulation of the contact degree of the interface of 2D/2D BiVO_4_/CsPbBr_3_ (BC) heterostructures by altering the growth time in the preparation process. The obtained sample with the highest interface contact ratio (*R*_eff_), 58.6%, demonstrated superior photocatalytic activity compared to samples with lower *R*_eff_ values of 52.6% and 40.5%. As for the edge-to-face mode of 2D HP/2D material interfaces, related studies are rarely reported. Recently, a close-contact edge-to-face Co_*x*_Bi_2-*x*_O_2_CO_3_/Cs_3_Bi_2_Br_9_ 2D/2D heterointerface was successfully synthesized by Teng et al.^96^ through an atoms-cosharing in situ growth strategy (Figure 6f). During the preparation process, Co_*x*_Bi_2-*x*_O_2_CO_3_ was first fabricated to serve as both substrate and template. Then CsBr and HBr precursors were introduced, enabling the in situ epitaxial growth of a Co_*x*_Bi_2-*x*_O_2_CO_3_/Cs_3_Bi_2_Br_9_ heterojunction through the formation of a Bi atom bridge. This fascinating edge-to-face architecture is attributed to the high lattice mismatch (*f* = 27.19%, where *f* is defined as (*b* – *a*)/*a*, with *a* and *b* the corresponding lattice spacings of the two components) of the Bi–Bi distance between the Co_*x*_Bi_2-*x*_O_2_CO_3_ (100) facet and the Cs_3_Bi_2_Br_9_ (100) facet, but good lattice matching between the Co_*x*_Bi_2-*x*_O_2_CO_3_ (002) facet and the Cs_3_Bi_2_Br_9_ (112) facet, with a small *f* of 0.75%. As a result, the strong coupling interaction between Co_*x*_Bi_2-*x*_O_2_CO_3_ and Cs_3_Bi_2_Br_9_ facilitates the formation of a direct Z-scheme heterojunction, holding powerful interfacial electric field for charge carrier transfer.

#### 3.2.3 0D HP/2D Material Interfaces

When the HP size is reduced to QDs that are coupled with 2D materials, 0D HP/2D material interfaces are formed. Through an in situ growth or self-assembly approach to load HP QDs on 2D materials, this construction strategy can leverage the excellent optoelectrical properties of HP QDs and the large specific surface area of 2D materials. Zhong et al.^97^ prepared a 0D-2D CsPbBr_3_–Co_3_O_4_ heterostructure via a facile self-assembly method by mixing CsPbBr_3_ QDs with Co_3_O_4_ nanosheets in solution. The huge surface energy of CsPbBr_3_ QDs drives them to attach to Co_3_O_4_ nanosheets with uniform distribution, leading to a rich heterointerface (Figure 6g). Upon light irradiation, the formed type II heterojunction motivates the transfer of electrons from the CB of CsPbBr_3_ to the CB of Co_3_O_4_, effectively facilitating CO_2_ photoreduction. Apart from nanosheets, 2D materials can also be present in a hierarchical flower shape. Wang et al.^72^ successfully fabricated 0D Cs_2_SnI_6_ QD/2D SnS_2_ nanosheets (Cs_2_SnI_6_/SnS_2_) by in situ construction of Cs_2_SnI_6_ QDs on SnS_2_ nanoflowers (Figure 6h). The low mismatch between the lattice spacing of the Cs_2_SnI_6_ [001] direction and the SnS_2_ [001] direction ensures the direct generation of Cs_2_SnI_6_ on the surface of SnS_2_. XPS characterization reveals the formation of S···Sn···I bonds in the Cs_2_SnI_6_/SnS_2_ heterostructure. This strong interface interaction induces a powerful IEF with boosted charge separation efficiency, and combined with the enhanced light absorption of the structure, this Cs_2_SnI_6_/SnS_2_ demonstrates remarkable photocatalytic CO_2_ reduction performance.

### 3.3 HP/2D Material Heterostructure Photocatalysts

2D materials constitute a broad category that includes members ranging from graphene, TMDs, and MXene to g-C_3_N_4_, metal oxides, and MOFs. The diversity and modifiable nature of HP and 2D materials allow for the development of multiple heterostructures with different charge transfer mechanisms. Depending to their photoelectric properties, 2D materials can serve as cocatalysts or the second semiconductor to couple with HPs and form heterojunctions including Schottky junctions, type I junctions, type II junctions, p-n junctions, Z-scheme junctions, and S-scheme junctions. Recently documented HP/2D material heterostructures based on the type of 2D material are summarized in Tables 1–6.

**Table 1:** HP/Graphene Heterostructure Photocatalysts

Photocatalyst	Type of heterostructure	Preparation strategy	Interface architecture	Application	Light source	Solution	Performance	Ref
CsPbBr_3_ QD/GO	Schottky junction	In situ growth	0D/2D	CO_2_ reduction	100 W Xe lamp with AM 1.5G filter	Ethyl acetate	CO_2_ to CO production rate of 58.7 μmol g^–1^	(100)
MAPbBr_3_/rGO-Pt	Schottky junction	In situ growth	3D/2D face to face	H_2_ evolution	Xe lamp (780 nm ≥ λ ≥ 400 nm)	CH_3_CN	H_2_ evolution rate of 3150 μmol h^–1^	(124)
CsPbBr_3_/GO	Schottky junction	In situ growth	3D/2D face to face	CO_2_ reduction	Xe lamp (λ > 400 nm)	Water	CH_4_ production rate of 18.6 μmol g^–1^ h^–1^	(125)
CsPbBr_3_/ZnO/GO	Schottky junction	Self-assembly	3D/2D face to face	CO_2_ reduction	Xe lamp (λ > 420 nm)	Water	CH_4_ yield rate of 6.29 μmol g^–1^ h^–1^	(126)
CsPbBr_3_/carboxyl-modified rGO	Schottky junction	Self-assembly	3D/2D face to face	CO_2_ reduction	Xe lamp	Water	total yields of CO and CH_3_OH products were 88.296 and 90.300 μmol g^–1^	(127)
CsPbBr_3_-Cu-RGO	Schottky junction	Ball milling	2D/2D face to face	CO_2_ reduction	Xe lamp	Water	CH_4_ and CO yield rates of 12.7 and 0.27 μmol g^–1^ h^–1^	(128)
Cs_2_AgBiBr_6_-Cu-RGO	Schottky junction	Ball milling	2D/2D face to face	CO_2_ reduction	Xe lamp	Water	CH_4_ and CO yield rates of 10.7 and 1.9 μmol g^–1^ h^–1^	(129)
MAPbI_3_/rGO	Schottky junction	Photoreduction	3D/2D face to face	H_2_ evolution	Xe lamp (λ > 420 nm)	MAPI_3_-saturated HI solution	H_2_ evolution rate of 93.9 μmol h^–1^	(101)
Cs_2_AgBiBr_6_/RGO	Schottky junction	Photoreduction	3D/2D face to face	H_2_ evolution	Xe lamp (λ > 420 nm)	Saturated HBr and H_3_PO_2_ solution	H_2_ evolution rate of 489 μmol g^–1^ within 10 h	(130)
Cs_2_SnBr_6_/rGO	Schottky junction	Photoreduction	3D/2D face to face	Organic synthesis	Xe lamp (λ > 400 nm)	MeCN solution containing 5 mM HMF	99.5% conversion of HMF and 88% DFF selectivity	(131)
α-Fe_2_O_3_/Amine-RGO/CsPbBr_3_	Solid-state Z-scheme	Solvent evaporation–deposition method	3D/2D face to face	CO_2_ reduction	Xe lamp (λ > 420 nm)	Water	Total yield of CH_4_, CO, and H_2_ of 469.16 μmol g^–1^ within 40 h	(102)
CsPbBr_3_-rGO/Bi_2_WO_6_	S-scheme	Self-assembly	3D/2D face to face	Pollutants degradation	Xe lamp (λ > 420 nm)	Norfloxacin solution	Norfloxacin degradation rate of 66.79% in 120 min	(132)

**Table 2:** HP/MXene Heterostructure Photocatalysts

Photocatalyst	Type of heterostructure	Preparation strategy	Interface architecture	Application	Light source	Solution	Performance	Ref
CsPbBr_3_/Ti_3_C_2_T_*x*_	Schottky junction	In situ growth	3D/2D face to face	CO_2_ reduction	Xe lamp (λ > 420 nm)	Ethyl acetate	CO and CH_4_ production rates of 26.32 and 7.25 μmol g^–1^ h^–1^	(105)
FAPbBr_3_QDs/Ti_3_C_2_	Schottky junction	In situ growth	0D/2D	CO_2_ reduction	Xe lamp	Water	CO and CH_4_ production rates of 283.41 and 17.67 μmol g^–1^ h^–1^	(133)
FAPbBr_3_/Ti_3_C_2_	Schottky junction	In situ growth	2D/2D face to face	CO_2_ reduction	Xe lamp	Water	CO yield of 93.82 μmol g^–1^ h^–1^	(134)
CsPbBr_3_/Ti_3_C_2_T_*x*_	Schottky junction	Impregnation deposition	3D/2D face to face	CO_2_ reduction	Xe lamp (λ > 400 nm)	Water	CH_4_ and CO generation rates of 3.5 and 42 μmol g^–1^ h^–1^	(135)
Cs_2_AgBiBr_6_/Ti_3_C_2_T_*x*_	Schottky junction	Self-assembly	3D/2D face to face	CO_2_ reduction	Xe lamp (λ > 400 nm)	Water	CO yield rate of 11.1 μmol g^–1^	(106)
MAPbI_3_/Ti_3_C_2_T_*x*_	Schottky junction	In situ growth	3D/2D face to face	H_2_ evolution	LED lamp (780 nm ≥ λ ≥ 370 nm)	MAPbI_3_-saturated HI–H_3_PO_2_ solution	H_2_ evolution rate of 63.6 μmol h^–1^	(107)
Ti_3_C_2_/NiO/CsPbI_3_	Z-scheme junction	Hydrothermal method	3D/2D face to face	Pollutants degradation	UV medium-pressure immersion lamp	Crystal-violet dye solution	Dye degradation rate of 92.8% within 90 min	(136)
Cs_3_Bi_2_Br_9_/Ti_3_C_2_T_*x*_	Schottky junction	In situ growth	3D/2D face to face	Organic synthesis	5 W blue LED	Toluene	conversion rate of 2121 μmol g^–1^ h^–1^ and 100% selectivity to benzaldehyde	(137)

**Table 3:** HP/g-C_3_N_4_ Heterostructure Photocatalysts

Photocatalyst	Type of heterostructure	Preparation strategy	Interface architecture	Application	Light source	Solution	Performance	Ref
CsPbBr_3_/g-C_3_N_4_	Type II	In situ growth	3D/2D face to face	CO_2_ reduction	Xe lamp equipped with AM 1.5 filter	–	CO_2_ to CO conversion rate of 5.52 μmol g^–1^ h^–1^	(138)
Cs_3_Sb_2_Br_9_/g-C_3_N_4_	Type II	In situ growth	3D/2D face to face	Organic synthesis	Xe lamp (λ > 420 nm)	Toluene	Total toluene conversion rate of 8346.8 μmol g^–1^ h^–1^	(139)
FAPbBr_3_/g-C_3_N_4_	Type II	In situ growth	3D/2D face to face	NO removal	Xe lamp	–	NO removal rate of 58% in 60 min	(109)
CsPbBrCl_2_/g-C_3_N_4_	Type II	In situ growth	3D/2D	Dye degradation	Xe lamp	40 m of 10^–5^ M aqueous solution of Eosin B dye	Degradation rate over 95% in 150 min	(140)
Cs_3_Bi_2_Br_9_/g-C_3_N_4_	Type II	Self-assembly	2D/2D face to face	Organic synthesis	Xe lamp	Toluene	Benzaldehyde formation rate of 4.53 mmol g^–1^h^–1^	(141)
Sb-substituted Cs_2_AgBiBr_6_/g-C_3_N_4_	Type II	Mechanical grinding	3D/2D face to face	Organic synthesis	Hg (Xe) lamp (λ > 420 nm)	2.0 mL of acetonitrile (ACN) with 70 μL of toluene	Benzaldehyde generation rate of 1088.0 mmol g^–1^h^–1^	(142)
Cs_3_Bi_2_Br_9_/g-C_3_N_4_	S-scheme	In situ growth	3D/2D	CO_2_ reduction	Xe lamp equipped with AM 1.5 filter	Isopropanol	CO production rate of 14.22 μmol g^–1^ h^–1^	(143)
CsPbBr_3_/S doped g-C_3_N_4_	S-scheme	In situ growth	3D/2D	CO_2_ reduction	Xe lamp (λ > 400 nm)	acetonitrile/deionized water (200:1 v:v, 5 mL)	CO production rate of 83.6 μmol g^–1^ h^–1^	(90)
CsPbBr_3_/porous g-C_3_N_4_	S-scheme	In situ growth	3D/2D	CO_2_ reduction	Xe lamp	Water	CO production rate of 30.39 μmol g^–1^ h^–1^	(144)
CsPbBr_3_/g-C_3_N_4_	S-scheme	Self-assembly	2D/2D face to face	CO_2_ reduction	Xe lamp equipped with AM 1.5 filter	10 mL of ethyl acetate and 30 μL of water	CH_4_ and CO production rates of 184.0 and105.2 μmol g^–1^	(92)
Cs_2_AgBiBr_6_@g-C_3_N_4_	S-scheme	Self-assembly	3D/2D face to face	CO_2_ reduction	Xe lamp	4 mL of ethyl acetate and 1 mL of methanol	CO_2_ reduction rate of 2.0 μmol g^–1^ h^–1^	(145)
Cs_2_AgBiBr_6_-g-C_3_N_4_	S-scheme	Mechanical grinding	3D/2D	CO_2_ reduction	Hg lamp (780 nm ≥ λ ≥ 285 nm)	Isopropyl alcohol	CH_4_ and CO production rates of 8.85 and 12.14 μmol g^–1^ h^–1^	(146)
Cs_3_Bi_2_I_9_/g-C_3_N_4_	Direct Z-scheme	Self-assembly	3D/2D face to face	Dye degradation	LED lamp	RhB solution	RhB degradation rate of 100% in 150 min	(147)
CsPbBr_3_/g-C_3_N_4_	S-scheme	Annealing	3D/2D	CO_2_ reduction	Xe lamp	–	CO_2_ to CO reduction rate of 42.8 μmol g^–1^ h^–1^	(110)

**Table 4:** HP/TMDs Heterostructure Photocatalysts

Photocatalyst	Type of heterostructure	Preparation strategy	Interface architecture	Application	Light source	Solution	Performance	Ref
MAPbI_3_/MoS_2_	Cocatalyst	In situ growth	3D/2D face to edge	H_2_ evolution	White light LED (380–780 nm, 450 mW/cm^2^)	MAPbI_3_-saturated solution	H_2_ evolution rate of 206.1 μmol h^–1^	(148)
MAPbBr_3-*x*_I_*x*_/MoSe_2_	Cocatalyst	In situ growth (antisolvent method)	3D/2D	H_2_ evolution	Visible light (>420 nm, 300 W)	HBr/HI/H_3_PO_2_	H_2_ production of 6.99 mmol g^–1^	(149)
(HA)PbI_4_/MoS_2_	Cocatalyst	In situ growth (reprecipitation method)	3D/2D	H_2_ evolution	Visible light (>350 nm, 300 W)	(HA)PbI_4_-saturated solution	H_2_ evolution rate of 3.9 mmol g^–1^ h^–1^	(150)
MA_1-*x*_FA_*x*_PbI_3_/MoS_2_	Cocatalyst	In situ growth (reprecipitation method)	3D/2D	H_2_ evolution	300 W xenon lamp	MAPbI_3_-saturated solution	H_2_ evolution rate of 2131 μmol g^–1^ h^–1^	(151)
MAPbI_3_/MoSe_2_	Type I	In situ growth (coprecipitation method)	3D/2D	H_2_ evolution	Visible light (>450 nm)	HI/H_3_PO_2_	H_2_ evolution rate of 552.93 μmol g^–1^ h^–1^	(73)
Cs_2_AgBiBr_6_/MoS_2_	Type I	In situ growth	3D/2D	H_2_ evolution	Visible light (>420 nm, 300 W)	HBr/H_3_PO_2_	H_2_ evolution rate of 87.5 μmol g^–1^ h^–1^	(113)
MAPbI_3_/MoS_2_	Type I	Self-assembly	3D/2D	H_2_ evolution	Visible light (>420 nm)	MAPbI_3_-saturated solution	H_2_ evolution rate of 339 μmol g^–1^ h^–1^	(152)
MAPbBr_3_/ReS_2_	Type I	In situ growth (antisolvent method)	3D/2D	Organic conversion	Visible light (>420 nm, 300 W)	3 mL of acetonitrile containing 200 μmol of BA	BAD and H_2_ production rates of about 1220 μmol g^–1^ h^–1^	(71)
MAPbI_3_/MoS_2_	Type II	In situ growth (coprecipitation method)	3D/2D	H_2_ evolution	Visible light (>420 nm, 280 W)	MAPbI_3_-saturated solution	H_2_ evolution rate of 30 000 μmol g^–1^ h^–1^	(57)
MAPbI_3_/MoS_2_	Type II	In situ growth (coprecipitation method)	3D/2D	H_2_ evolution	Visible light (>420 nm, 300 W)	MAPbI_3_-saturated solution	H_2_ evolution rate of 13.6 mmol g^–1^ h^–1^	(69)
Cs_3_Bi_2_I_9_/MoS_2_ QDs	Type II	Self-assembly	3D/0D	H_2_ evolution	Visible light (>420 nm)	Ethanol solution, HI, H_3_PO_2_	H_2_ evolution rate of 6.09 mmol g^–1^ h^–1^	(91)
FAPbBr_3_/MoS_2_	Type II	In situ growth (antisolvent method)	3D/2D	H_2_ evolution and organic conversion	Visible light (>420 nm, 300 W)	3 mL of acetonitrile containing 0.2 mmol of aromatic alcohols	H_2_ evolution rate of 1150 μmol g^–1^ h^–1^ and benzyl aldehyde (BAD) generation rate of 1040 μmol g^–1^ h^–1^	(153)
CsPbBr_3_/MoS_2_	Type II	Self-assembly	3D/2D	CO_2_ reduction	Visible light (>420 nm, 300 W)	5 mL of ethyl acetate and 20 μL of deionized water	Conversion rates of CO_2_ to CH_4_ and CO of 12.8 and 25.0 μmol g^–1^ h^–1^	(114)
FAPbBr_3_/WS_2_	Z-scheme	Self-assembly	2D/2D	Organic conversion	150 W Xe lamp with AM 1.5 G filter	0.1 mmol of benzyl alcohol and 2.5 mL of benzotrifluoride	Conversion rate and selectivity of benzyl alcohol to benzoic acid of 75.9% and 99%	(70)

**Table 5:** HP/Metal Oxides or Bismuth-Based Materials Heterostructure Photocatalysts

Photocatalyst	Type of heterostructure	Preparation strategy	Interface architecture	Application	Light source	Reaction solution	Performance	Ref
CsPbBr_3_/TiO_2_	S-scheme	In situ growth	3D/2D	CO_2_ reduction and benzyl alcohol oxidation	300 W Xe lamp	Benzotrifluoride and benzyl alcohol	Production rates of CO 78.06 μmol g^–1^ h^–1^ and BD 1.77 mmol g^–1^ h^–1^	(154)
CsPbBr_3_/TiO_2_	Type II	In situ growth (antisolvent method)	3D/2D	Toluene oxidation	Visible light (>420 nm, 300 W)	Toluene and acetonitrile	Toluene oxidation rate of 10200 μmol g^–1^ h^–1^ with 85% selectivity	(115)
FAPbBr_3_/WO_3_	Z-scheme	In situ growth (antisolvent method)	0D/2D	Benzyl oxidation	Simulated solar illumination	–	Benzoic conversion rate over 65% with 90% selectivity	(75)
CsPbBr_3_/Co_3_O_4_	Type II	Self-assembly	0D/2D	CO_2_ reduction	Visible light (>400 nm, 300 W)	Water	CO and CH_4_ production rates of 35.4 and 29.2 μmol g^–1^ h^–1^	(97)
Cs_3_Bi_2_I_9_/CeO_2_	Z-scheme	Self-assembly	2D/2D	CO_2_ reduction	300 W Xe lamp	Water	Total CH_4_ and CO production rate of 238.21 μmol g^–1^	(155)
Cs_3_Bi_2_Br_9_/V_2_O_5_	Z-scheme	Mechanical mixing and calcination	3D/2D	CO_2_ reduction	300 W Xe lamp	Water	CO production rate of 37.8 μmol g^–1^ h^–1^	(156)
CsPbBr_3_/CuCo_2_O_4_	Z-scheme	Self-assembly	0D/2D	CO_2_ reduction	300 W Xe lamp with AM 1.5 G filter	Ethyl acetate	CH_4_ yield rate of 285.93 μmol g^–1^	(157)
CsPbBr_3_/Bi_2_WO_6_	Z-scheme	In situ growth	0D/2D	CO_2_ reduction	Visible light (>400 nm)	Ethyl acetate and water	Total CH_4_ and CO yield of 503 μmol g^–1^	(54)
Cs_3_Bi_2_Br_9_/Bi_2_WO_6_	S-scheme	Self-assembly	0D/2D	CO_2_ reduction	300 W Xe lamp with AM 1.5 G filter	Toluene	CO yield rate of 220.1 μmol g^–1^ h^–1^	(158)
Cs_2_AgBiBr_6_/Bi_2_WO_6_	Z-scheme	In situ growth	0D/2D	CO_2_ reduction	300 W Xe lamp with AM 1.5 G filter	Ethyl acetate and isopropanol alcohol	CO yield rate of 42.19 μmol g^–1^ h^–1^	(159)
CsPbBr_3_/BiVO_4_	S-scheme	In situ growth	2D/2D	CO_2_ reduction	300 W Xe lamp	Water	CO production rate of 103.5 μmol g^–1^ with 97% selectivity	(95)
CsPbBr_3_/BiOCl	S-scheme	Self-assembly	2D/2D	CO_2_ reduction	300 W Xe lamp	Ethyl acetate and water	CO and CH_4_ production rates of 34.72 and 3.47 μmol g^–1^ h^–1^	(76)
CsPbBr_3_/Bi_3_O_4_Br	Z-scheme	Self-assembly	2D/2D	CO_2_ reduction	300 W Xe lamp	Water	CO production of 387.57 μmol g^–1^	(93)
Cs_3_Bi_2_Br_9_/BiOBr	S-scheme	In situ growth	3D/2D	Toluene oxidation	300 W Xe lamp	Toluene and acetonitrile	Toluene conversion rate of 22.5% with 96.2% selectivity	(117)
CsPbBr_3_/BiOI/TiO_2_@PAN	S-scheme	In situ growth	3D/2D	Pollutants degradation	300 W Xe lamp	RhB solution	RhB removal rate of 99.5% in 90 min	(160)
CsPbBr_3_/Bi_2_MoO_6_	S-scheme	Self-assembly	3D/2D	Pollutants degradation	300 W Xe lamp with AM 1.5 G filter	RhB solution	RhB removal rate of 95.8% in 90 min	(78)
CsPbBr_3_/BiFeO_3_	Z-scheme	In situ growth	3D/2D	CO_2_ reduction	300 W Xe lamp (>420 nm)	Water	CO yield rate of 53.1 μmol g^–1^ h^–1^	(79)
Cs_2_AgBiBr_6_/H_2_WO_4_	S-scheme	In situ growth	2D/2D	CO_2_ reduction	300 W Xe lamp	Ethyl acetate	CH_4_ yield rate of 22.6 μmol g^–1^ h^–1^	(161)
CsPbBr_3_/T-SrTiO_3_	Type II	In situ growth	2D/2D	CO_2_ reduction	300 W Xe lamp	Water	CO production rate of 120.2 μmol g^–1^ h^–1^	(162)

**Table 6:** HP/Other Materials Heterostructure Photocatalysts

Photocatalyst	Type of heterostructure	Preparation strategy	Interface architecture	Application	Light source	Reaction solution	Performance	Ref
CsPbBr_3_/NiAl-LDH	Z-scheme	Self-assembly	3D/2D	CO_2_ reduction	300 W Xe lamp	Ethyl acetate and water	CO yield of 17.24 μmol g^–1^ h^–1^	(122)
CsPbBr_3_/NiCo-LDH	Type II	Self-assembly	0D/2D	CO_2_ reduction	300 W Xe lamp (>420 nm)	Acetonitrile and water	CO yield of 204.4 μmol g^–1^ h^–1^ with 100% selectivity	(121)
CsPbBr_3_/CoAl-LDH	S-scheme	Self-assembly	3D/2D	CO_2_ reduction	300 W Xe lamp (>420 nm)	Ethyl acetate and water	Total CO and CH_4_ generation rate of 59.5 μmol g^–1^ h^–1^	(80)
CsPbBr_3_/Ni-MOF	Type II	Self-assembly	1D/2D	CO_2_ reduction	300 W Xe lamp (>420 nm)	Ethyl acetate and water	CO yield of 81.0 μmol g^–1^ h^–1^	(53)
Cs_2_AgBiBr_6_/Ni-MOF	S-scheme	Self-assembly	2D/2D	CO_2_ reduction	300 W Xe lamp	Ethyl acetate	Generation rates of CO and CH_4_ of 58.85 and 15.43 μmol g^–1^ h^–1^	(94)
CsPbBr_3_/CuTCPP MOF	Type II	In situ growth	0D/2D	CO_2_ reduction	300 W Xe lamp with AM 1.5 G filter	Ethyl acetate and water	Total CO and CH_4_ generation rates of 59.5 and 47.2 μmol g^–1^ h^–1^	(163)
CsPbBr_3_/SnS_2_	Z-scheme	Self-assembly	3D/2D	CO_2_ reduction	Xe lamp (300–800 nm)	Water	CO yield of 1.98 μmol g^–1^ h^–1^	(164)
Cs_2_SnI_6_/SnS_2_	Type II	In situ growth	0D/2D	CO_2_ reduction	Xe lamp (>420 nm)	Methanol and water	CH_4_ yield of 6.09 μmol g^–1^ h^–1^	(72)
Cs_3_Bi_2_Br_9_/In_4_SnS_8_	S-scheme	In situ growth	0D/2D	CO_2_ reduction	300 W Xe lamp (>420 nm)	Water	CO yield of 57.3 μmol g^–1^ h^–1^	(98)
CsPbBr_3_/CdIn_2_S_4_	Type II	Self-assembly	0D/2D	CO_2_ reduction	300 W Xe lamp (>420 nm)	Ethyl acetate and water	CO yield of 9.5 μmol g^–1^ h^–1^	(123)
CsPbBr_3_/PbSe	Type I	Self-assembly	0D/2D	CO_2_ reduction	300 W Xe lamp (>400 nm)	Water and isopropanol	CO yield of 322.4 μmol g^–1^ h^–1^	(165)

#### 3.3.1 2D Materials Act as Cocatalysts

##### 3.3.1.1 HP/Graphene Heterostructure Photocatalysts

**Figure 7:**
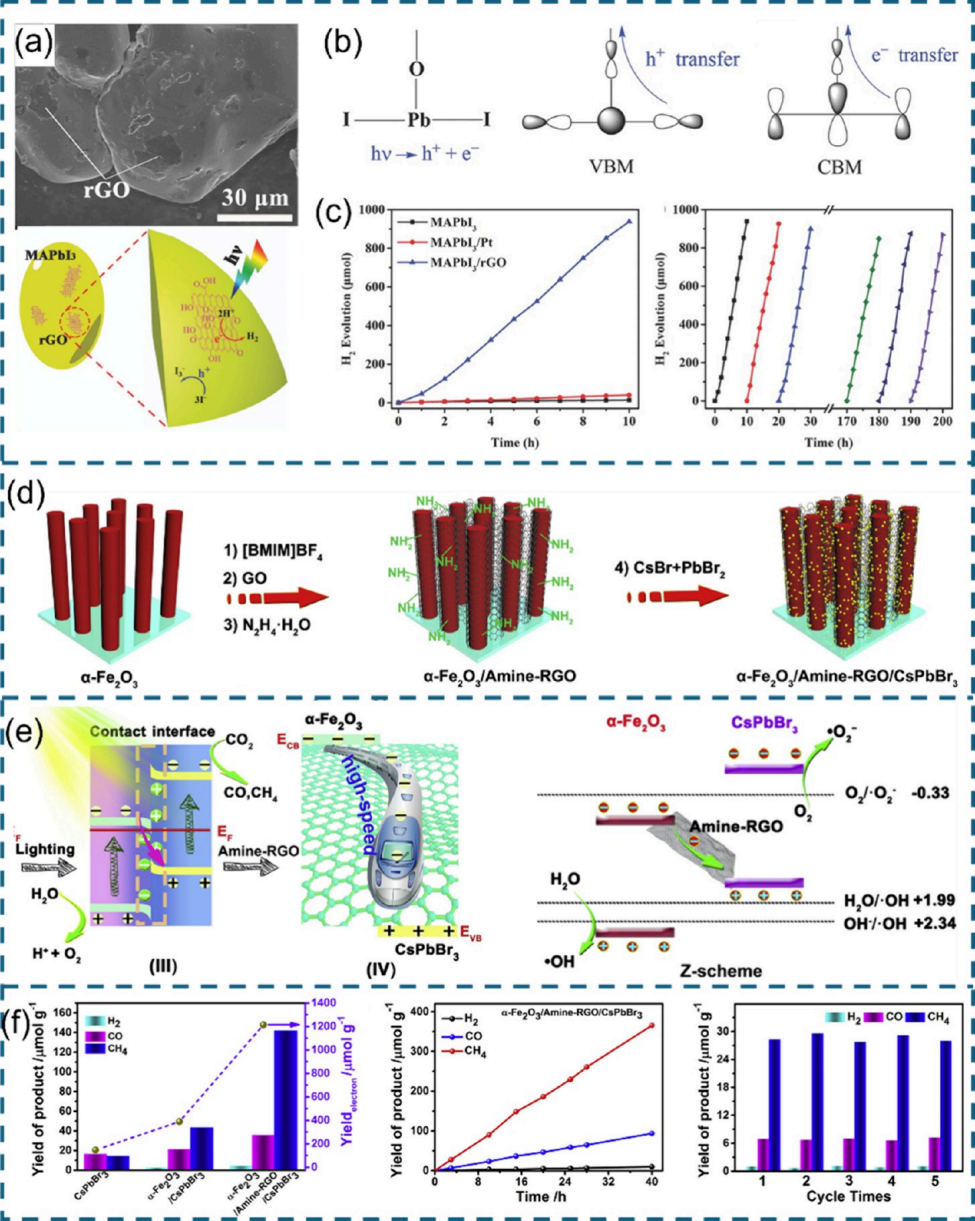
(a) SEM image and illustration of MAPbI_3_/rGO. (b) Scheme of atom arrangement and orbital character around the Pb site of a Pb–O–C bridge between rGO and MAPbI_3_. (c) H_2_ evolution performance of MAPbI_3_/rGO. Reproduced with permission from ref (101). Copyright 2018, John Wiley and Sons. (d) Design illustration of α-Fe_2_O_3_/Amine-rGO/CsPbBr_3_. (e) Illustration of the charge transfer mechanism of Z-scheme α-Fe_2_O_3_/Amine-rGO/CsPbBr_3_ and (d) corresponding CO_2_ reduction performance. Reproduced with permission from ref (102). Copyright 2020, Cell Press.

Graphene is a 2D allotrope of carbon with a honeycomb structure. The sp^2^-hybridized carbon atoms in graphene endow it with remarkable charge carrier mobility, electrical conductivity, and optical transmittance.^99^ Graphene oxide (GO) is a derivative of graphene with rich oxygen functional groups and exhibits a desirable solution dispersibility. Reduced graphene oxide (rGO) can be obtained through the reduction of GO. The exceptional electrical conductivity and compatibility with solution-based processes make GO and rGO desirable materials for constructing heterostructures with HP semiconductors. In a typical HP/graphene photocatalyst composite, HP serves as an excellent light absorber, while GO/rGO acts as a cocatalyst to immediately receive and transport photoexcited electrons from the CB of HP, which significantly enhances the separation and transfer of charge carriers, as well as the surface redox reactions. The first example of an HP/graphene photocatalyst is the CsPbBr_3_ QD/graphene oxide (CsPbBr_3_ QD/GO) reported by Xu et al. in 2017 for CO_2_ photoreduction. Through a simple antisolvent method, CsPbBr_3_ QDs are grown in situ on GO with an even distribution. Owing to the lower Fermi level of GO compared to the CB of CsPbBr_3_ QD, photogenerated electrons swiftly transfer to GO, thus facilitating fast separation of photogenerated electron–hole pairs and suppressed charge recombination.^100^ Subsequently, Wu et al.^101^ reported a MAPbI_3_/reduced graphene oxide composite (MAPbI_3_/rGO) for H_2_ evolution from HI solution. In a facile photoreduction preparation process, GO powders are reduced to rGO and deposited on the surface of MAPbI_3_ under visible light irradiation (Figure 7a). FTIR and XPS characterizations suggest bonding between MAPbI_3_ and rGO through the formation of a Pb–O–C linkage. In MAPbI_3_, electron–hole pairs are generated by the PbI_3_ framework. The VBM of PbI_3_ is primarily characterized by the sigma antibonding interactions between the Pb 6s and I 5p orbitals, while the CBM is dominated by the pi antibonding interactions between the Pb 6p and I 5p orbitals. In the Pb–O–C bridge, the Pb–O bond involves Pb 6p–O 2p overlap at the CBM energy region and Pb 6s–O 2p overlap at the VBM energy region. Given that the Pb 6p–O 2p overlap is stronger than the Pb 6s–O 2p overlap, electron transfer via the Pb–O–C bridge to the rGO unit occurs faster for electrons near the CBM than for holes near the VBM (Figure 7b). Benefiting from the rapid electron transfer pathway, MAPbI_3_/rGO exhibits an outstanding H_2_ evolution performance (93.9 μmol h^–1^) compared to pristine MAPbI_3_ (1.4 μmol h^–1^) (Figure 7c). In addition to coupling with HPs to form a Schottky junction, rGO can also serve as an electron mediator in an all-solid-state Z scheme heterojunction between HP and another semiconductor. Jiang et al.^102^ used α-Fe_2_O_3_, amine-functionalized rGO (Amine-rGO), and CsPbBr_3_ to construct an α-Fe_2_O_3_/ Amine-rGO/CsPbBr_3_ Z-scheme heterojunction (Figure 7d). The staggered energy band structure and a big difference in Fermi level between α-Fe_2_O_3_ and CsPbBr_3_ promote the formation of an IEF at the interface. Upon light irradiation, Amine-rGO with high conductivity and electron affinity can facilitate the electron extraction from α-Fe_2_O_3_ and provide a speedway for subsequent electron transport to CsPbBr_3_, contributing to a strengthened Z-scheme transfer at the interface (Figure 7e). As a consequence of efficient charge carrier separation, the remaining electrons at the CB of CsPbBr_3_ and holes at the VB of α-Fe_2_O_3_ with robust redox potentials result in excellent CO_2_ photoreduction performance (Figure 7f).

##### 3.3.1.2 HP/MXene Heterostructure Photocatalyst

MXene is an emerging class of 2D materials with the general formula of M_*n*+1_X_*n*_T_*n*_ (*n* = 1–4), where M is an early transition metal, X represents carbon and/or nitrogen, and T refers to surface terminal groups.^103^ Benefiting from its excellent conductivity, tunable structure properties, and facile fabrication methods,^104^ MXene has raised interest in photocatalysis applications through coupling with semiconductors for enhanced photocatalytic performance. Ti_3_C_2_ is a typical MXene with metal-like behavior and has been successfully incorporated with HPs to construct the Schottky junction. The CB positions of most HPs are higher than the Fermi level of Ti_3_C_2_, meaning that photoexcited electrons will transfer from the CB of HPs to Ti_3_C_2_ and hence boost the charge carrier separation. Pan et al.^105^ first reported a CsPbBr_3_/MXene composite in 2019 through in situ growth of CsPbBr_3_ nanocrystals (CsPbBr_3_ NCs) on exfoliated Ti_3_C_2_T_*x*_ nanosheets. Owing to the efficient charge transfer between CsPbBr_3_ and MXene nanosheets, the optimal CsPbBr_3_/MXene sample demonstrates increased photocurrent generation and exhibits superior CH_4_ and CO production rates of 7.25 μmol g^–1^ h^–1^ and 26.32 μmol g^–1^ h^–1^, respectively, compared to bare CsPbBr_3_ NCs (<4.4 μmol g^–1^ h^–1^). MXene typically possesses a negatively charged surface because of the surface functional groups, which indicates that positively charged metal cations are prone to adhering to their surface. Based on this character, Zhang et al.^106^ synthesized a lead-free Cs_2_AgBiBr_6_/MXene heterostructure via a facile self-assembly method. The positive zeta potential of Cs_2_AgBiBr_6_ NCs (+86.7 mV) and negative zeta potential of Ti_3_C_2_T_*x*_ nanosheets (−39.0 mV) result in a strong electrostatic attraction between their surfaces, ensuring a stable and intimate combination of the heterostructure. As a result, the obtained Cs_2_AgBiBr_6_/Ti_3_C_2_T_*x*_ heterostructure behaves promoted charge carrier transfer and separation, with a remarkable photoelectron consumption yield of 50.6 μmol g^–1^ h^–1^ for photocatalytic CO_2_ reduction. What’s more, MXene as an effective cocatalyst for HPs in H_2_ photocatalytic evolution has also been demonstrated by Li et al. using MAPbI_3_–Ti_3_C_2_T_*x*_ NS composite in the MAPbI_3_-saturated HI–H_3_PO_2_ solution. The uniformly dispersed Ti_3_C_2_T_*x*_ nanosheets (NSs) in the reaction solution function as a nucleation matrix with a vast surface area for MAPbI_3_, which not only augments the dispersion of MAPbI_3_ crystals but also significantly intensifies the dynamic interplay between MAPbI_3_ and the Ti_3_C_2_T_*x*_ NSs. This, in turn, expedites the electron transfer process from photoexcited MAPbI_3_ to Ti_3_C_2_Tx NSs. Consequently, the charge separation efficiency in the MAPbI_3_–Ti_3_C_2_T_*x*_ NS composite is greatly boosted, leading to a 43 times enhancement of the H_2_ evolution rate (63.6 μmol h^–1^) compared to that of MAPbI_3_.^107^

#### 3.3.2 2D Materials Act as Photocatalysts

##### 3.3.2.1 HP/g-C_3_N_4_ Heterostructure Photocatalyst

**Figure 8:**
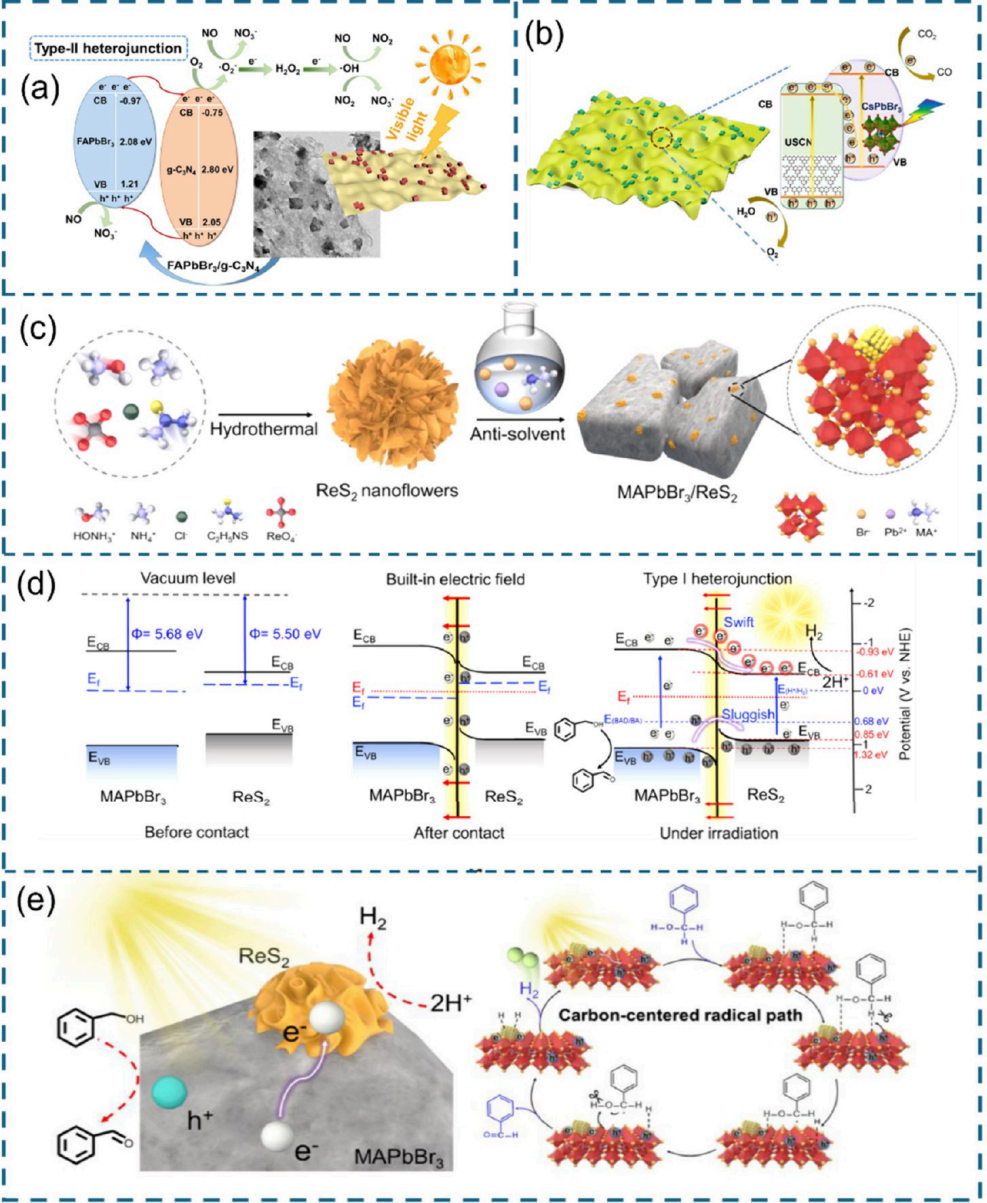
(a) Photocatalytic NO reduction mechanism of the FAPbBr_3_/g-C_3_N_4_ type II heterostructure. Reproduced with permission from ref (109). Copyright 2022, Elsevier. (b) Photocatalytic CO_2_ reduction mechanism of the CsPbBr_3_/g-C_3_N_4_ S-scheme heterostructure. Reproduced with permission from ref (90). Copyright 2022, Elsevier. (c) Fabrication of MAPbBr_3_/ReS_2_. (d) Schematic illustration of band structures and the charge transfer of MAPbBr_3_/ReS_2_. (e) Mechanism of photocatalytic conversion of biomass-derived alcohols to aldehydes and H_2_ using MAPbBr_3_/ReS_2_. Reproduced with permission from ref (71). Copyright 2024, Elsevier.

g-C_3_N_4_ exhibits a graphene-like structure, featuring periodically arranged heptazine rings that are formed through the p^2^ hybridization of C and N atoms. Its inherently thin atomic structure facilitates the formation of a stacking layered semiconductor through van der Waals forces.^108^ With intriguing properties such as suitable band gap (∼2.7 eV) and band positions, facile fabrication and modification process, and exceptional stability, g-C_3_N_4_ has gained significant popularity as a photocatalyst in recent decades. Unfortunately, solely g-C_3_N_4_ shows limited photocatalytic activity because of unsatisfactory utilization of visible light and severe recombination of photoinduced electrons and holes. Hence, taking advantage of HPs and g-C_3_N_4_ to construc HP/g-C_3_N_4_ heterostructures is a desirable strategy for better photocatalysis performance. Based on the band structures of HP and g-C_3_N_4_, the commonly formed interfacial heterojunction type could be type II or S-scheme. Xie et al.^109^ fabricated FAPbBr_3_/g-C_3_N_4_ through an in situ growth method at room temperature. XPS characterization reveals the shift of binding energy of Pb 4f and Br 3d, suggesting a strong interaction between FAPbBr_3_ and g-C_3_N_4_. Besides, the higher CB and VB positions of FAPbBr_3_ compared to g-C_3_N_4_ lead to the formation of a type II heterojunction. Upon light irradiation, the photoexcited electrons on the CB of FAPbBr_3_ will immediately migrate to the CB of g-C_3_N_4_, and the photogenerated holes on the VB of g-C_3_N_4_ will spontaneously transfer to the VB of FAPbBr_3_, hence achieving efficient spatial separation of charge carriers (Figure 8a). In another study, Bian et al.^110^ designed a core–shell (m-CN@CsPbBr_3_) heterostructure with CsPbBr_3_ nanoparticles encapsulated by 2D g-C_3_N_4_. The strong interfacial interaction between CsPbBr_3_ and g-C_3_N_4_ owing to the intensely wrapped structure leads to the formation of IEF where the electron depletion region on the surface of g-C_3_N_4_ and electron accumulation region on the surface of CsPbBr_3_. The IEF drives the photogenerated electrons on the CB of g-C_3_N_4_ combining with the holes on the VB of CsPbBr_3_ in an S-scheme pathway, which facilitates charge carrier separation and retains electrons and holes on strong redox potentials. Accordingly, such an S-scheme heterojunction demonstrates an outstanding CO_2_-to-CO yield of 42.8 μmol g^–1^ h^–1^. Modifications of g-C_3_N_4_ can further enhance the photocatalytic activity of the HP/g-C_3_N_4_ heterostructure. Zhao et al.^90^ used sulfur-doped ultrathin g-C_3_N_4_ (USCN) as the substrate for CsPbBr_3_ (CPB) to construct the CPB/USCN composite. The sulfur doping endows USCN with a broader light absorption edge compared to that of pristine g-C_3_N_4_, further improving the light-harvesting ability of the CPB/USCN heterostructure. Moreover, the doped sulfur in USCN enhances its coupling effect with Pb in CPB and ensures solid chemical and electronic interaction at the interface. This facilitates an S-scheme transfer route of photogenerated charge carriers which is proved by in situ XPS spectroscopy. Hence, photogenerated electrons with robust reduction potential are accumulated on the CB of CPB, and photogenerated holes holding sufficient oxidation potential are retained on the VB of USCN, finally contributing to the CPB/USCN 16 times higher CO_2_ photoreduction rate (83.6 μmol g^–1^ h^–1^) than pristine CsPbBr_3_ (Figure 8b).

##### 3.3.2.2 HP/TMDs Heterostructure Photocatalyst

TMDs form with the chemical formula MX_2_, where the metallic atom layer M (e.g., Mo and W) is connected to two layers of chalcogen atoms X (S and Se). The neighboring layers of TMDs are coupled via van der Waals forces and form a 2D layered structure.^111^ Owing to variations in the coordination geometry of the transition metal atoms, TMDs exhibit different structure phases such as 2H (trigonal prismatic), 1T (octahedral), and 1T′ (distorted octahedral). Among them, the 2H phase holds semiconductor character and generally has a narrow band gap (<2.4 eV), demonstrating a capability for solar spectrum utilization and strong charge carrier generation.^22,112^ Additionally, the robust stability makes TMDs resist extremely highly acidic saturated solutions of HPs, which extends the preparation methods and reaction environments of HPs-based heterostructures. In recent years, studies have gradually proven that TMDs could be an ideal partner for HPs for high photocatalysis performance. Generally, the CB positions of TMDs being lower than those of HPs makes them electron acceptors in the HP/TMDs composite, forming type I or II heterojunctions. For instance, Zhang et al.^113^ fabricated a Cs_2_AgBiBr_6_/MoS_2_ heterostructure with improved photocatalytic activity for H_2_ evolution. The former type I heterojunction drives the electrons and holes generated by Cs_2_AgBiBr_6_ transfer to the CB and VB of MoS_2_, followed by fast redox reactions on the MoS_2_ surface. As a result, Cs_2_AgBiBr_6_/MoS_2_ performs a remarkable H_2_ evolution rate of 87.5 μmol g^–1^ h^–1^ in an aqueous HBr solution, which is 20 times that of pure CsAgBiBr_6_ (4.3 μmol g^–1^ h^–1^). In a recent study, ReS_2_ was embedded on the MAPbBr_3_ surface to construct the MAPbBr_3_/ReS_2_ photocatalyst by Shan et al.^71^ The special embedded structure ensures intense interface contact and facilitates a strong built-in electric field between MAPbBr_3_ and ReS_2_ (Figure 8c). More importantly, the low work function of ReS_2_ matches well with the energy band of MAPbBr_3_, which can accelerate electron transfer from the CB of MAPbBr_3_ to the CB of ReS_2_ via downward band bending at the interface. At the same time, the upward VB of ReS_2_ lags the h^+^ transfer from MAPbBr_3_, thereby empowering efficient separation of photoinduced electron/hole pairs (Figure 8d). Accordingly, this type I heterojunction demonstrates impressive benzaldehyde and H_2_ production rates of 1180 and 1220 μmol g^–1^ h^–1^, respectively (Figure 8e). In addition, type II CsPbBr_3_/MoS_2_ was fabricated by Wang et al.^114^ by anchoring CsPbBr_3_ nanocrystals on 2D MoS_2_ nanosheets. DFT calculations reveal that Pb–S covalent bonds are formed between MoS_2_ and CsPbBr_3_ and act as tunnels for direct electron transfer. Based on this, electrons on the S atom of MoS_2_ will migrate and accumulate on the CsPbBr_3_ surface, resulting in a strong IEF to promote the transfer of electrons and holes. Consequently, such efficient charge carrier separation leads to exceptional photocatalytic CO_2_ reduction performance.

##### 3.3.2.3 HP/Metal Oxide Heterostructure Photocatalyst

**Figure 9:**
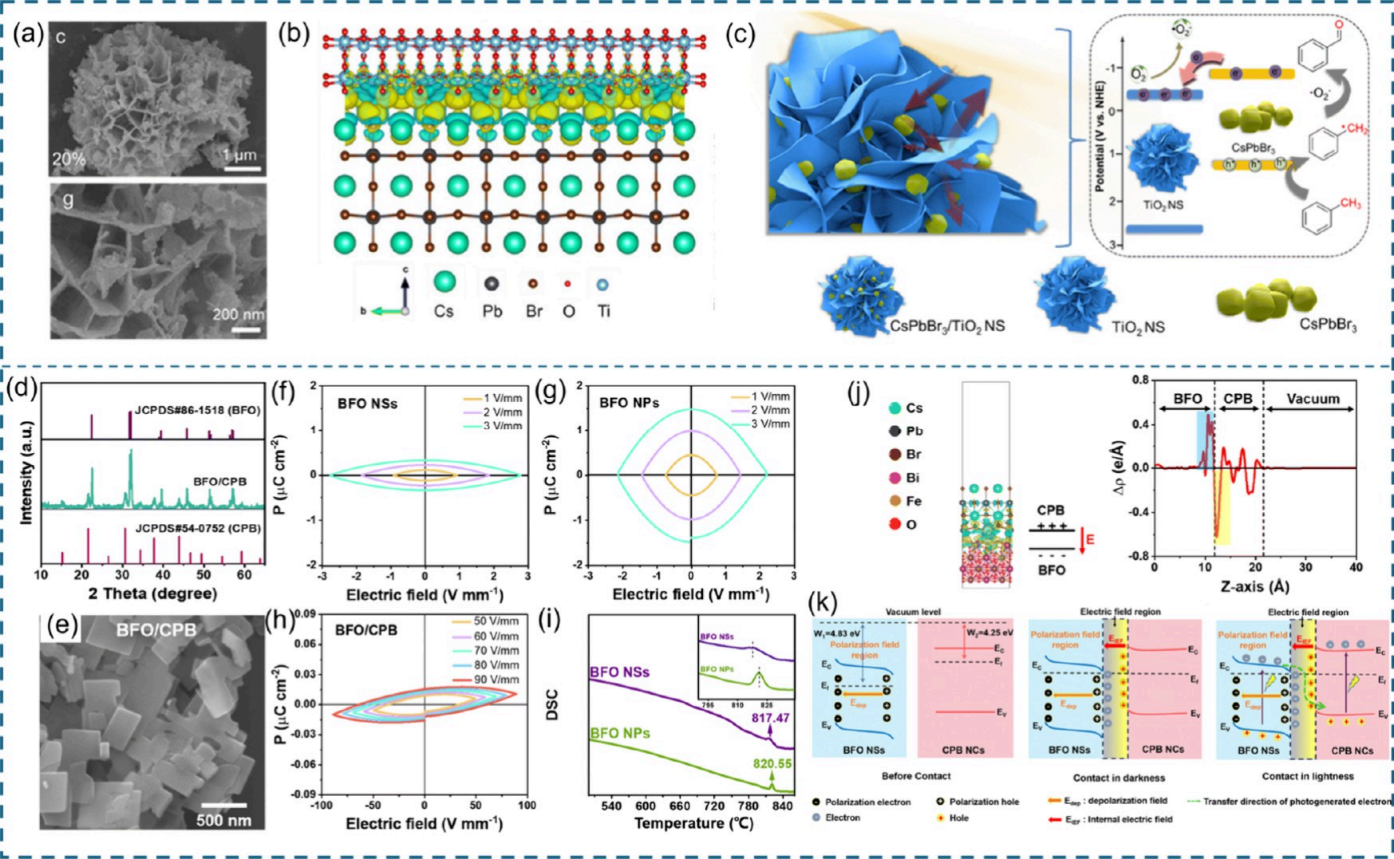
(a) SEM images of CsPbBr_3_/TiO_2_. (b) Charge density difference at the CsPbBr_3_/TiO_2_ interface. (c) Schematic illustration of photocatalytic toluene oxidation over CsPbBr_3_/TiO_2_. Reproduced with permission from ref (115). Copyright 2023, the Royal Society of Chemistry. (d) XRD pattern and (e) SEM image of BiFeO_3_/CsPbBr_3_. Polarization electric field hysteresis loops of (f, g) BiFeO_3_ and (h) BiFeO_3_/CsPbBr_3_. (i) Differential scanning calorimetry of CsPbBr_3_ and BiFeO_3_. (j) Charge density difference at the BiFeO_3_/CsPbBr_3_ interface. (k) Illustration of the charge transfer mechanism in the BiFeO_3_/CsPbBr_3_ heterojunction before and after contact. Reproduced with permission from ref (79). Copyright 2024, the Royal Society of Chemistry.

Metal oxides were the first documented and are the most extensively researched materials in the field of photocatalysis. The construction of metal oxides with 2D morphology can effectively improve the surface area and the number of active sites, offering an ideal platform for the loading of HPs. Interestingly, controlling the dimensions of metal oxides is always accompanied by the change of exposure facet, which may alter the surface charge transfer due to the facet-dependent electronic structure. Therefore, desirable photocatalyst heterostructures with optimized interfacial electronic structures could be attained through reasonable facet modulation. Based on this, Wang et al.^74^ employed TiO_2_ nanosheets with respectively exposed (101) (T-101) and (001) (T-001) facets as substrates for loading CsPbBr_3_ QDs. More significant shifts of the binding energy of Ti 2p and Pb 4f in T-101/CsPbBr_3_ indicate a stronger interfacial electron redistribution than in T-001/CsPbBr_3_. Besides, DFT calculation shows that the differences in work functions between CsPbBr_3_ and T-101 are larger than those between CsPbBr_3_ and T-001, which means a more powerful driving force for electron transfer at the interface of T-101/CsPbBr_3_. Ultimately, T-101/CsPbBr_3_ demonstrates extraordinary photocatalytic CO_2_ reduction performance with a high CO yield of 12.5 μmol g^–1^ h^–1^, which is 5.6 times higher than that of T-001/CsPbBr_3_. In addition to facet modulation, morphology regulation on metal oxides can also enhance the overall photocatalytic performance by optimizing the distribution of HPs on the surface of metal oxides. For example, Yi et al.^115^ designed a hierarchical nanoflower structure consisting of 2D TiO_2_ nanoflakes for supporting CsPbBr_3_ particles. Through an in situ antisolvent precipitation process, CsPbBr_3_ particles are uniformly distributed on the surface of TiO_2_ nanoflowers. The formation of the CsPbBr_3_/TiO_2_ heterostructure prevents the agglomeration of CsPbBr_3_ particles, contributing to a large surface area and excellent adsorption capacity for toluene molecules (Figure 9a). Moreover, the close contact between CsPbBr_3_ and TiO_2_ facilitates an intensively interacted interface with electron migration to TiO_2_ from CsPbBr_3_ (Figure 9b). Consequently, a type II heterojunction is formed, showing promoted charge carrier transfer and separation. Thanks to the cocontributions of enhanced light harvesting, efficient charge separation, and impressive gas adsorption, the well-designed hierarchical CsPbBr_3_/TiO_2_ achieves a remarkable toluene oxidation rate of 10200 μmol g^–1^ h^–1^, which is 20 times higher than single CsPbBr_3_ (Figure 9c). The tunable facet and morphology also allow metal oxides to be a suitable platform to investigate the size effect of HPs. Wang et al.^75^ loaded FAPbBr_3_ NCs and FAPbBr_3_ QDs respectively on the flower-shape WO_3_ nanosheets (f-WO_3_) with (002) facet exposure to study the influence of quantum confinement effect on the photocatalytic oxidation of benzyl alcohol. They found that the photocatalysis products present significant differences when the size of FAPbBr_3_ reduced from NCs to QDs, attributed to the different reaction rates. In particular, the W–O bonds in WO_3_ could be broken under illumination becoming oxygen and subsequently converted to ^•^O_2_^–^ via accepting photoinduced electrons from FAPbBr_3_. This oxygen departure process leads to oxygen vacancy generation. The higher oxygen vacancy concentrations detected in FAPbBr_3_ QDs/f-WO_3_ compared to FAPbBr_3_ NCs/f-WO_3_ may also result in different conversion rates.

##### 3.3.2.4 HP/Bismuth-Based Materials Heterostructure Photocatalyst

Generally speaking, bismuth-based materials used in photocatalysis can be divided into two classes: bismuth oxyhalides (BiOX, X = Cl, Br, or I) and bismuth-based polyoxometalates (Bi_2_WO_6_, Bi_2_MoO_6_, or BiVO_4_). The key compositions of most of them are the [Bi_2_O_2_]^+^ layers connected through weak van der Waals force, and this configuration makes them intrinsically present 2D morphology.^116^ Favored by the properties of suitable band gap, visible light responsiveness, and satisfactory stability, bismuth-based compounds have been deemed as ideal coupling semiconductors with HPs. Typical HP/bismuth-based material heterostructure are reported as CsPbBr_3_/BiOCl,^76^ Cs_3_Bi_2_Br_9_/BiOBr,^117^ Cs_3_Bi_2_I_9_/Bi_2_WO_6_,^118^ CsPbBr_3_/Bi_2_MoO_6_^78^ and so on (Table 5). As concluded from the reported studies, since the energy band positions (CB and VB) and the Fermi levels of HPs are generally higher than those of bismuth-based semiconductors, an S-scheme heterojunction will be formed at the interface of HP/bismuth-based material composite, with the electrons on the CB of bismuth-based semiconductor consumed by the holes on the VB of HP. Hence, the remaining electrons on the CB of HP and holes on the VB of bismuth-based semiconductors possessing robust redox potentials can significantly boost the photocatalytic performance. Excitingly, some bismuth-based materials with a non-centrosymmetric structure represented as BiFeO_3_ not only possess narrow band gap (2.0–2.2 eV) but also exhibit extraordinary ferroelectric properties. The ferroelectric domain can induce the rearrangement of dipoles in BiFeO_3_, resulting in a polarized field that drives the charge separation.^119^ To this end, a novel BiFeO_3_/CsPbBr_3_ heterostructure was constructed through loading CsPbBr_3_ NCs on BiFeO_3_ nanosheets in a recent study.^79^ The construction of the BiFeO_3_/CsPbBr_3_ heterojunction enhances the synergistic effect of spontaneous ferroelectric polarization field and interfacial electric field, providing a powerful driving force for the transfer and spatial separation of photogenerated charge carriers (Figure 9f-k). Consequently, this Z-scheme heterojunction presents an impressive photocatalytic CO production rate of 53.1 μmol g^–1^ h^–1^, which is 6.4 times that of blank CsPbBr_3_.

##### 3.3.2.5 HP/Other 2D Materials Heterostructure Photocatalyst

Other semiconductors with a 2D morphology such as LDHs and MOFs are also engaged with HPs to construct heterostructures in recent years. LDHs have typically layered 2D structures. The easily changeable metal cations and anions of LDHs render an adjustable electronic structure. Besides, the alkaline nature of LDHs enables them satisfactory affinity for CO_2_ adsorption.^120^ Notably, most LDHs own relatively low valence bands, which makes them have high oxidant capability and suitable for constructing heterostructures with other semiconductors. Moreover, by modification of the preparation methods, the obtained LDHs can show a hierarchical flower-like structure, which provides a large surface area and abundant active sites. Reported HP/LDH heterostructures like CsPbBr_3_/CoAl-LDH,^80^ CsPbBr_3_/NiCo-LDH,^121^ and CsPbBr_3_/NiAl-LDH^122^ possess S-scheme charges pathway with electrons in the CB of LDHs recombine with holes in the VB of CsPbBr_3_, exhibiting significantly higher CO_2_ photocatalytic performance compared to the single component. For 2D MOFs, Ni-based MOFs (Ni-MOF) are regarded as suitable loading substrates for HPs due to their large surface area, good light adsorption capacity, and stability. It is reported that Ni-MOF as a support for CsPbBr_3_ can significantly improve the CO_2_ uptake capacity from ∼2.5 cm^3^ g^–1^ (pristine CsPbBr_3_) to 15.3 cm^3^ g^–1^ (Ni-MOF/CsPbBr_3_) because the abundant pores and surface sites can promote CO_2_ adsorption. In contrast to bare CsPbBr_3_, the PL intensity of Ni-MOF/CsPbBr_3_ shows a dramatic decrease when changing the atmosphere from Ar to CO_2_, indicating the accelerated transfer of electrons to adsorbed CO_2_ with the aid of Ni-MOF.^53^ Recently, transition metal chalcogenides as emerging 2D layered materials have attracted tremendous interest, owing to their suitable energy structure, broad light absorption, and extensive surface area. Researchers have started to try to combine them with HPs for a better photocatalysis performance. For example, the 2D/0D CdIn_2_S_4_/CsPbBr_3_ with type II heterojunction at the interface exhibits high charge separation efficiency. Integrating with numerous catalytic active sites provided by CsIn_2_S_4_, this composite achieved enhanced CO_2_ photocatalytic reduction performance.^123^ In another study, In_4_SnS_8_ nanosheets composed of nanoflower serve as an ideal substrate for Cs_3_Bi_2_Br_9_ QDs, providing large surface area and rich active sites for CO_2_ adsorption and reduction. The construction of In_4_SnS_8_/Cs_3_Bi_2_Br_9_ is beneficial to the formation of intermediates and reduces the energy barrier of the CO_2_ photocatalytic reduction reaction.^98^

## 4 Interfacial Charge Carrier Mechanism

The behavior of the charge carriers (especially charge separation and separation) in photocatalysts plays a crucial role in the photocatalysis process and directly determines the photocatalytic performance. Through the construction of heterointerfaces, HP/2D material photocatalysts demonstrate superior photocatalytic activity in comparison to single-component HP or 2D materials. This enhancement is closely related to the efficient transfer and separation of photoinduced electrons and holes, as well as favorable charge carrier dynamics. Thus, understanding the mechanisms underlying the enhanced performance of the HP/2D material heterostructures is essential.

### 4.1 Enhanced Interfacial Charge Carrier Behavior in HP/2D Material Heterostructures

#### 4.1.1 Accelerating Charge Transfer

**Figure 10:**
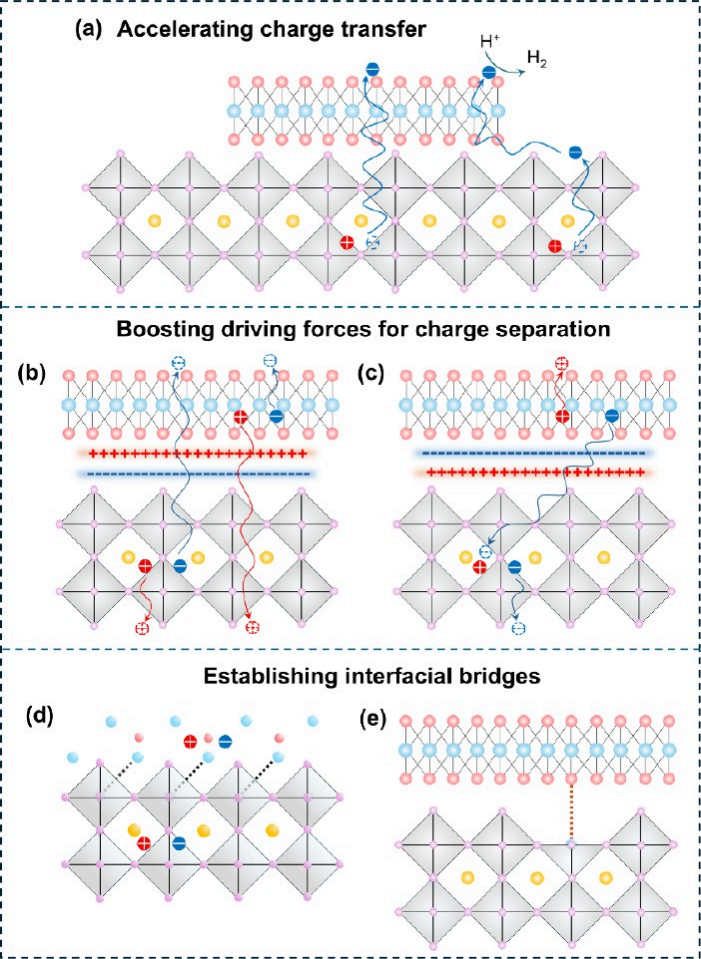
Schematic illustration of charge separation and transfer modes at the interface of the HP/2D material. (a) The 2D material extracts electrons from the HP. (b, c) A BIEF formed at the interface of the 2D material and the HP facilitates charge separation and transfer. (d, e) A bridge is established between the 2D material and the HP through identical or different atomic elements.

Upon light irradiation, electrons and holes are generated in HPs and will diffuse from the bulk to the surface. Unfortunately, large amounts of them suffer from severe recombination before reaching the surface. The random movement of photogenerated charge carriers on the surface also leads to their insufficient utilization. Faced with this problem, 2D materials with excellent conductivity attached to the surface of the HPs can effectively promote the transfer of charge carriers. In this case, as shown in Figure 10a, photoexcited electrons in the bulk or on the surface will directionally transfer to 2D materials swiftly. Moreover, the minimal thickness of 2D materials significantly reduces the charge transfer distance and contributes to immediate surface reactions. 2D materials such as MoS_2_, GO, and Ti_3_C_2_ have been reported to exhibit comparable or even better electron conductivity compared to noble metals.^91,107,130^ Noteworthily, the integration of 2D materials can offer an additional nonradiative channel for electron transfer from HP to 2D materials, which has been proven by the quenching phenomenon of photoluminescence (PL) intensity. As a consequence, the accelerated electron transfer and restrained charge recombination result in a higher photocatalytic activity.

#### 4.1.2 Boosting Driving Forces for Charge Separation

The difference in electronic structure between HP and 2D materials can induce the electron transfer at the interface and result in the formation of the built-in electric field, which will drive the transfer and separation of photogenerated electrons and holes, as illustrated in Figure 10b and c. When HP and 2D materials contact each other, electron transfer will occur to attain the alignment between their Fermi levels. During this process, the one with a lower Fermi level will accept electrons from another one with a higher Fermi level, and finally, an electron accumulation region and an electron depletion region are formed at the surface of the former and latter, respectively. The charge difference at the interface serves as a powerful electric field and controls the transfer pathway of photogenerated charge carriers. Assuming that HP holds more negative CB and VB and lower Fermi levels than those of a 2D material, electrons from the 2D material will migrate to the HP, resulting in a negatively charged layer on the surface of the HP and a positively charged layer on the surface of 2D material. Consequently, a BIEF pointing from the 2D material to the HP will form. Upon light stimulation, the BIEF will drive electrons in the CB of the HP downward to transfer to the CB of the 2D material, while holes in the VB of the 2D material move upward to the VB of the HP (Figure 10b). Conversely, if the CB, VB, and Fermi levels of the HP are all higher than those of the 2D material, then electrons will transfer from the HP to the 2D material and a BIEF directing from the HP to the 2D material will be created, boosted by electron depletion at the HP side and electron accumulation at the 2D material side. Under illumination, electrons in the CB of the 2D material will recombine with holes in the VB of the HP. Ultimately, electrons in the CB of the HP and holes in the VB of the 2D material will be maintained (Figure 10c).

#### 4.1.3 Establishing Charge Highways

Establishing interfacial bridges could achieve an extraordinary close-contact interface, which significantly steers the charge transfer and separation. Building interfacial bridges involves bond formation in the vertical direction of the interface. This connection mode can diminish the distance and energy barrier of charge transfer, providing a superior alternative to a bridge-free interface.^166^ A typical bond-bridge interface could be formed through an in situ growth process if there are common elements in the composition of HP and 2D material (Figure 10d). For example, a Co_0.97_Bi_1.03_O_2_CO_3_/Cs_3_Bi_2_Br_9_ heterointerface forms via bridges between Bi atoms inducing strong chemical and electronic coupling interaction, which can facilitate efficient channels for electron transfer from Co_0.97_Bi_1.03_O_2_CO_3_ to CsBi_2_Br_9_.^96^ In another similar example, an in situ formed Cs_2_SnI_6_/SnS_2_ heterostructure with Sn atoms cosharing exhibiting extremely rapid charge separation between Cs_2_SnI_6_ and SnS_2_.^72^ Also, interfacial bridges may form between different atoms if surface vacancies exist (Figure 10e). It is predicted through theoretical calculations that the introduction of Br vacancies at the CsSnBr_3_/SnS_2_ interface could significantly enhance the BIEF with charge density difference improved by 10 times compared to the interface without Br vacancies. This remarkable enhancement could be attributed to the formation of interfacial Sn–S bonds which provide high-speed charge transfer pathways from SnS_2_ to the CsSnBr_3_ surface.^167^

### 4.2 Studying Charge Carrier Separation with Space-Resolved Characterizations

In order to gain a direct understanding of the charge transfer direction and the spatial distribution at the interface of HP/2D material heterostructures, some advanced techniques such as in situ XPS, SPV, and KPFM are performed to decipher the spatial patterns of charge carrier behavior.

**Figure 11:**
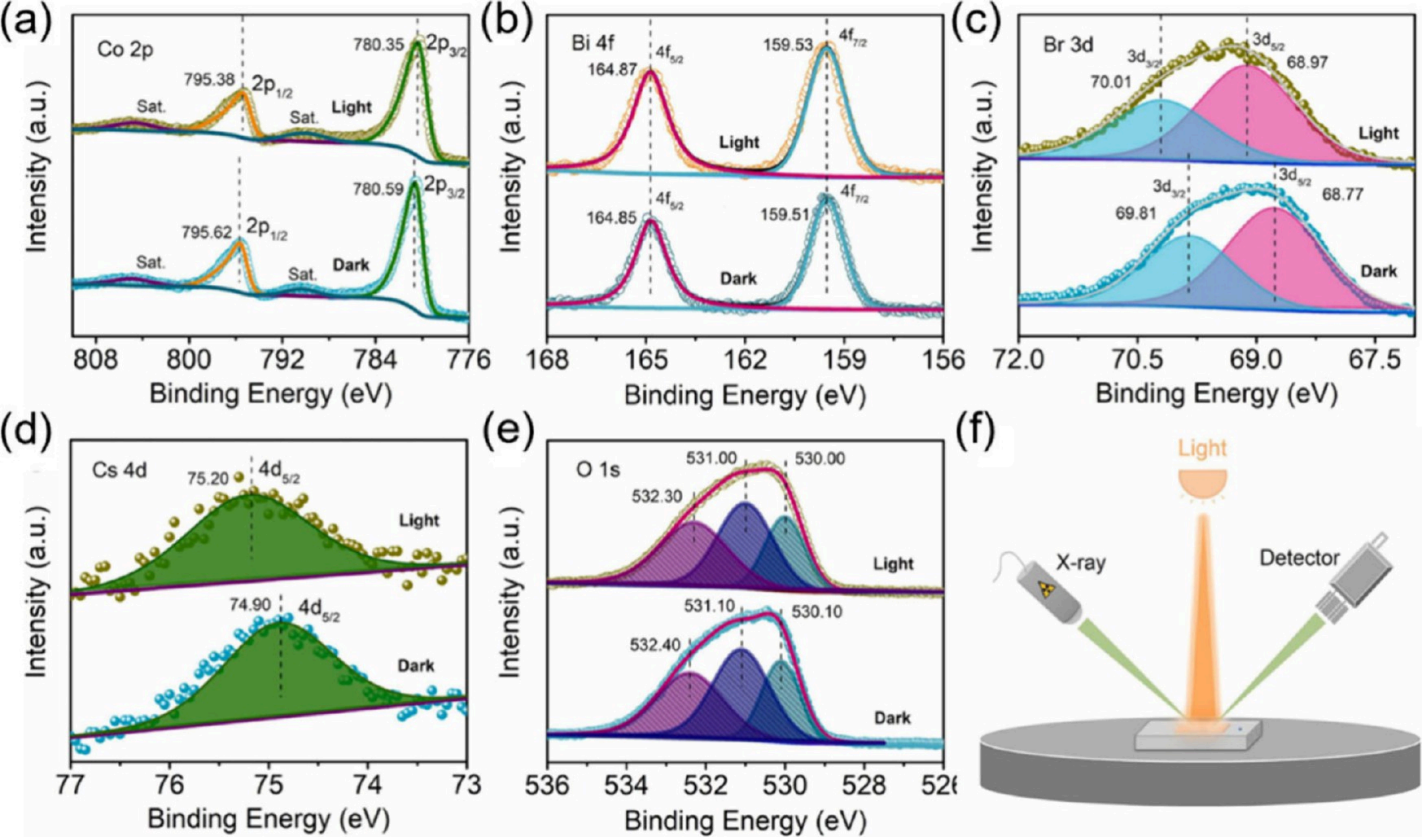
(a–e) In situ XPS spectra of different elements. (f) Schematic illustration of the in situ XPS test. Reproduced with permission from ref (96). Copyright 2023, Elsevier.

XPS can provide the surface chemistry of photocatalysts by probing the variation of the electronic density. Interactions between the strong Coulomb effect of the nucleus and the electrostatic shielding effect of the surrounding electrons lead to a specific binding energy of the inner electrons within an atom. Hence the charge transfer around the atom will induce electron density changes and be reflected by the variation in binding energy. Specifically, electron loss will lead to a decrease in electron density with higher binding energy, whereas electron acquisition will result in an increase in electron density and lower binding energy.^168^ Therefore, through monitoring the variation of the element-binding energy, XPS is expected to describe the charge transfer in a photocatalyst heterostructure system. Interfacial charge transfer may occur when two semiconductors come into contact under light irradiation. With the aid of XPS measurement, the charge transfer route or the type of heterojunction can be determined. For example, the XPS spectra in dark conditions show that the binding energies of Co 2p and O 1s in Co_0.97_Bi_1.03_O_2_CO_3_/Cs_3_Bi_2_Br_9_ heterostructure move toward higher values, and the Br 3d shift to lower binding energy simultaneously, compared to pristine Co_0.97_Bi_1.03_O_2_CO_3_ and Cs_3_Bi_2_Br_9_, respectively. While under illumination, negative shifts of the binding energy of Co 2p and O 1s are observed in the heterojunction. At the same time, Br 3d and Cs 4d show positive binding energy shifts (Figure 11). This opposite binding energy shift direction with and without light irradiation demonstrates that photoinduced electrons transfer to Co_0.97_Bi_1.03_O_2_CO_3_ from Cs_3_Bi_2_Br_9_ through an S-scheme pathway.^96^

**Figure 12:**
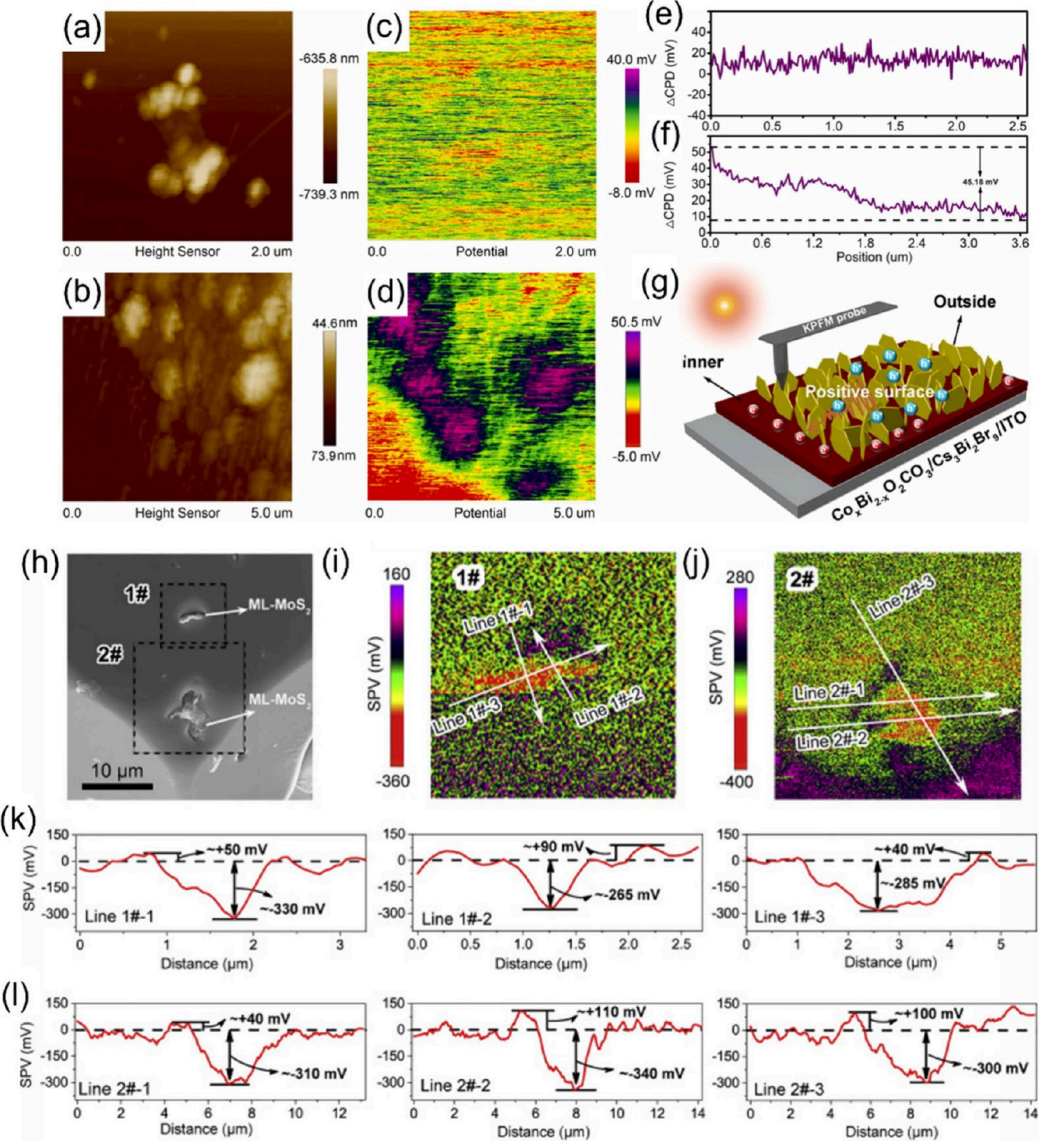
(a, c) AFM and (b, d) SPV images of Co_0.97_Bi_1.03_O_2_CO_3_ and Co_0.97_Bi_1.03_O_2_CO_3_/Cs_3_Bi_2_Br_9_. (e, f) SPV differences. (g) Schematic illustration of the distribution of photoinduced charges. Reproduced with permission from ref (96). Copyright 2023, Elsevier. (h) SEM image of MAPbI_3_/MoS_2_. (i, j) SPVM images of different regions and (k, l) corresponding SPV signal lines. Reproduced with permission from ref (69). Copyright 2020, Cell Press.

The spatial transfer and separation of photoexcited charges will induce photovoltage on the surface of the photocatalyst. This induced surface photovoltage (SPV) further causes variation in the contact potential difference (CPD) on the surface of the sample. Kelvin probe force microscopy (KPFM), consisting of a Kelvin probe and atomic force microscopy (AFM), can directly measure the light-induced change of the contact potential difference (ΔCPD) and image the SPV signal on a nanometer scale with high spatial resolution.^169^ This enables a direct presentation of charge separation and transfer in the space. Continuing the above case of Co_0.97_Bi_1.03_O_2_CO_3_/Cs_3_Bi_2_Br_9_ heterostructure, it exhibits much higher SPV changes before and after illumination with ΔCPD = +45.18 mV, in comparison to bare Co_0.97_Bi_1.03_O_2_CO_3_ with almost no SPV changes, which indicates significant enhancement of charge transfer and separation in the heterostructure (Figure 12a–g). It can also be concluded that photoinduced holes accumulate at the surface of Cs_3_Bi_2_Br_9_ nanosheets as they dominate the heterostructure surface, further proving that charge separation and transfer are driven via an S-scheme route.

SPV imaging can also be achieved by SPV microscopy (SPVM) by showing the difference between the KPFM scans before and after illumination. SPVM images can reflect the accumulation of photoexcited holes and electrons with positive and negative signals, respectively, thereby offering a clear visualization of the spatial distribution of photogenerated charge carriers. For instance, the distribution of photoexcited electrons and holes at the surface of MAPbI_3_/MoS_2_ heterostructure is clarified by SPVM.^69^ The obvious color contrast at the surface confirms that electrons and holes are concentrated on MoS_2_ (negative SPV) and MAPbI_3_ (positive SPV), respectively (Figure 12h–j). Moreover, the SPV distribution along line scans in different directions shows variation signals between positive and negative values, demonstrating effective charge separation and transfer at the MAPbI_3_/MoS_2_ heterointerface promoted by strong BIEF (Figure 12k,l).

### 4.3 Revealing Charge Carrier Dynamics with Time-Resolved Techniques

Photocatalysts under illumination could emit fluorescence through the radiative recombination of photoinduced electrons and holes, which can be recorded by PL spectroscopy. The lower PL intensity generally reflects the suppressed radiative recombination of electron–hole pairs in the photocatalyst. However, PL could not provide detailed information about charge carrier dynamics. To this end, time-resolved photoluminescence (TRPL) is an ideal option as it can describe the lifetime of photoexcited charge carriers and the process of their separation and transfer. By measuring the time between photoexcitation and photon emission of an electron, one can express a PL decay process. TRPL decay spectra are usually fitted with a multiexponential model (eq 3), and the average lifetime of photoexcited electrons can be calculated by eq 4:^170,171^
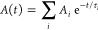
3
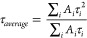
4where *A* refers to the amplitude and *t* represents the corresponding PL lifetime.

**Figure 13:**
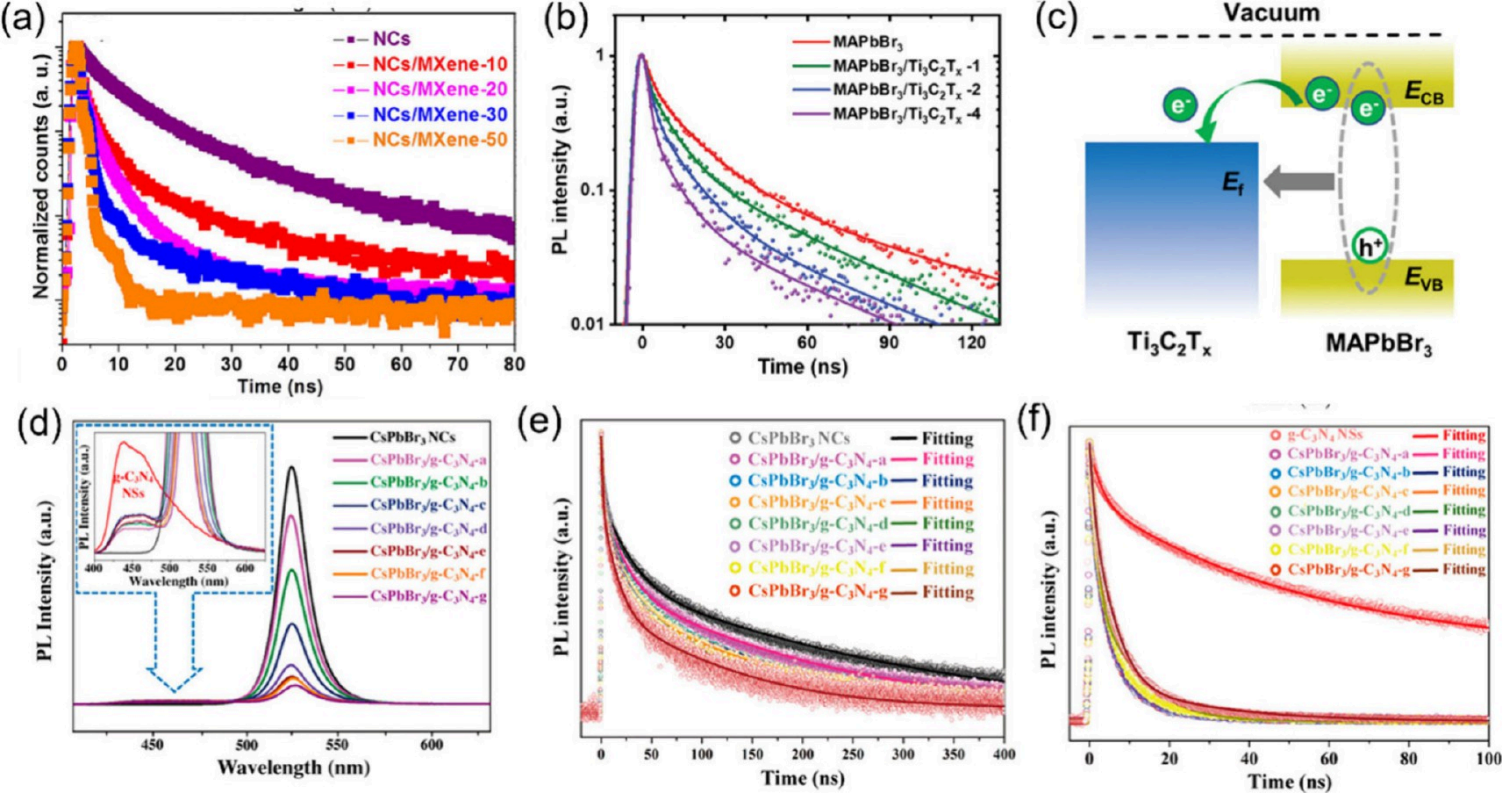
(a) PL decay spectra of CsPbBr_3_ and CsPbBr_3_/MXene. Reproduced with permission from ref (105). Copyright 2019, American Chemical Society. (b) TRPL spectra of MAPbBr_3_ and MAPbBr_3_/Ti_3_C_2_T_*x*_ heterostructures. (c) Band alignment and charge transfer between MAPbBr_3_ and Ti_3_C_2_T_*x*_. Reproduced with permission from ref (172). Copyright 2020, Wiley-VCH. (d) PL spectra of g-C_3_N_4_ and CsPbBr_3_/g-C_3_N_4_. (e) TRPL spectra of CsPbBr_3_ and CsPbBr_3_/g-C_3_N_4_ at their PL peaks at 525 nm. (f) TRPL spectra of g-C_3_N_4_ and CsPbBr_3_/g-C_3_N_4_ at their PL peaks at 440 nm. Reproduced with permission from ref (138). Copyright 2022, American Chemical Society.

Construction of the heterostructure could lead to a reduced or prolonged TRPL lifetime compared to that of the original single photocatalyst. In general, a shortened TRPL lifetime may be attributed to the accelerated charge transfer via a nonradiative pathway. A lengthened TRPL lifetime could result from the inhibited charge recombination in the photocatalyst.^171^ When HPs are coupled with 2D materials such as rGO and MXene that act as cocatalysts, shortened charge carrier lifetimes are usually observed. A typical example is that when CsPbBr_3_ NCs are loaded on Ti_3_C_2_T_*x*_ nanosheets, the average PL lifetime of obtained CsPbBr_3_/Ti_3_C_2_T_*x*_ samples presents significantly continuous reduction (from 4.1 to 0.21 ns) with the increase of Ti_3_C_2_T_*x*_ amount compared to pristine CsPbBr_3_ (18.8 ns), which is in accordance with the severe quenching of PL intensity (Figure 13a). The shortened PL lifetime of CsPbBr_3_/Ti_3_C_2_T_*x*_ indicates fast photoinduced electron transfer from CsPbBr_3_ to Ti_3_C_2_T_*x*_ through an efficient nonradiative channel.^105^ Another similar study also demonstrated this phenomenon of shortened PL decay and proposed that more MXene nanosheets can provide large amounts of surface groups for the growth of MAPbBr_3_ NCs, enabling strong interfacial interaction and thereby facilitating the charge transfer^172^ (Figure 13b,c). Integrating HPs with 2D materials to construct a type II heterojunction could also induce the reduction of PL lifetime. Chen et al.^138^ used different excitation wavelengths to compare the PL intensity and decay processes between CsPbBr_3_/g-C_3_N_4_ and a single component (CsPbBr_3_ or g-C_3_N_4_). With the increased incorporation amount of g-C_3_N_4_, the average PL lifetime of CsPbBr_3_/g-C_3_N_4_ shows a gradual decline compared to those of CsPbBr_3_ and g-C_3_N_4_ (Figure 13d–f). In this type II heterojunction, photogenerated electrons transfer from the CB of g-C_3_N_4_ to the CB of CsPbBr_3_, and holes transfer from the VB of CsPbBr_3_ to g-C_3_N_4_. Hence, the shortened average PL lifetime suggests facilitated electron and hole transfer. Based on the TRPL results, the authors calculated the electron transfer rate constant (*K*_et_) and hole transfer rate constant (*K*_ht_) and found that *K*_ht_ is almost 1 order lower than *K*_et_, which means that the hole transfer process in the CsPbBr_3_/g-C_3_N_4_ heterostructure could be the rate-limit step for the photocatalysis reaction. However, some other type II heterojunctions such as CsBi_2_Br_9_/g-C_3_N_4_^141^ and CsPbBrCl_2_/g-C_3_N_4_^140^ are reported that exhibit prolonged PL decay time than the sole component with suppressed charge recombination. Such phenomena of PL time variation discrepancy (shorten or prolong) can be also observed in S-scheme HP/2D material heterostructures. The elongation of PL lifetime may suggest that the recombination of photoinduced electrons and holes occurs at the trap state at the catalyst surface so that before recombination, these charge carriers have a higher possibility to participate in the photocatalytic reaction. In essence, the analysis and interpretation of TRPL decays should be conducted with caution and are better combined with other characterizations and specific photocatalysis performance.

Transient absorption spectroscopy (TAS) is a powerful time-resolved technique for studying the charge carrier dynamics of the photocatalysis process. Compared to TRPL with detection resolution at the nanoseconds time scale, TAS can measure both emissive and nonemissive exciton processes within the femtoseconds to picoseconds time scale.^173^ TAS employs a probe–pump instrumentation group and absorption spectroscopy to obtain data on the photoexcited charge behaviors. In a typical procedure, the photocatalyst sample is first excited to the excited state by using a pump pulse with high-energy femtosecond monochromatic laser, then a probe pulse (low-energy white pulse) will record the relaxation process of excitons from the excited state back to the ground state.^166^ The time delay between the two pumps is adjusted through a delay line. Through the conversion of sample transmittance (*T*), the optical absorption value (*A*) detected by the spectrometer is calculated as follows:^174^
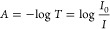
5where *I*_0_ and *I* are the pulse intensities of the probe before and after passing through the sample, respectively. Subsequently, the change in absorption spectrum (Δ*A*) between the excited state with a pump pulse (*A*_w_) and the ground state without a pump pulse (*A*_wo_) can be expressed as follows:
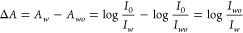
6The produced Δ*A* signal is an overlapping spectrum consisting of a ground state bleaching signal (GSB), an excited state absorption signal (ESA), and a simulated emission signal (SE). The GSB monitors the shift in electrons as they transition from their stable ground state to an energized excited state. Conversely, the ESA observes the advancement of electrons that are already excited to a more energetic, higher-level excited state. Additionally, the SE characterizes the relaxation process in which electrons return from the excited state to the ground state, simultaneously releasing fluorescence.

**Figure 14:**
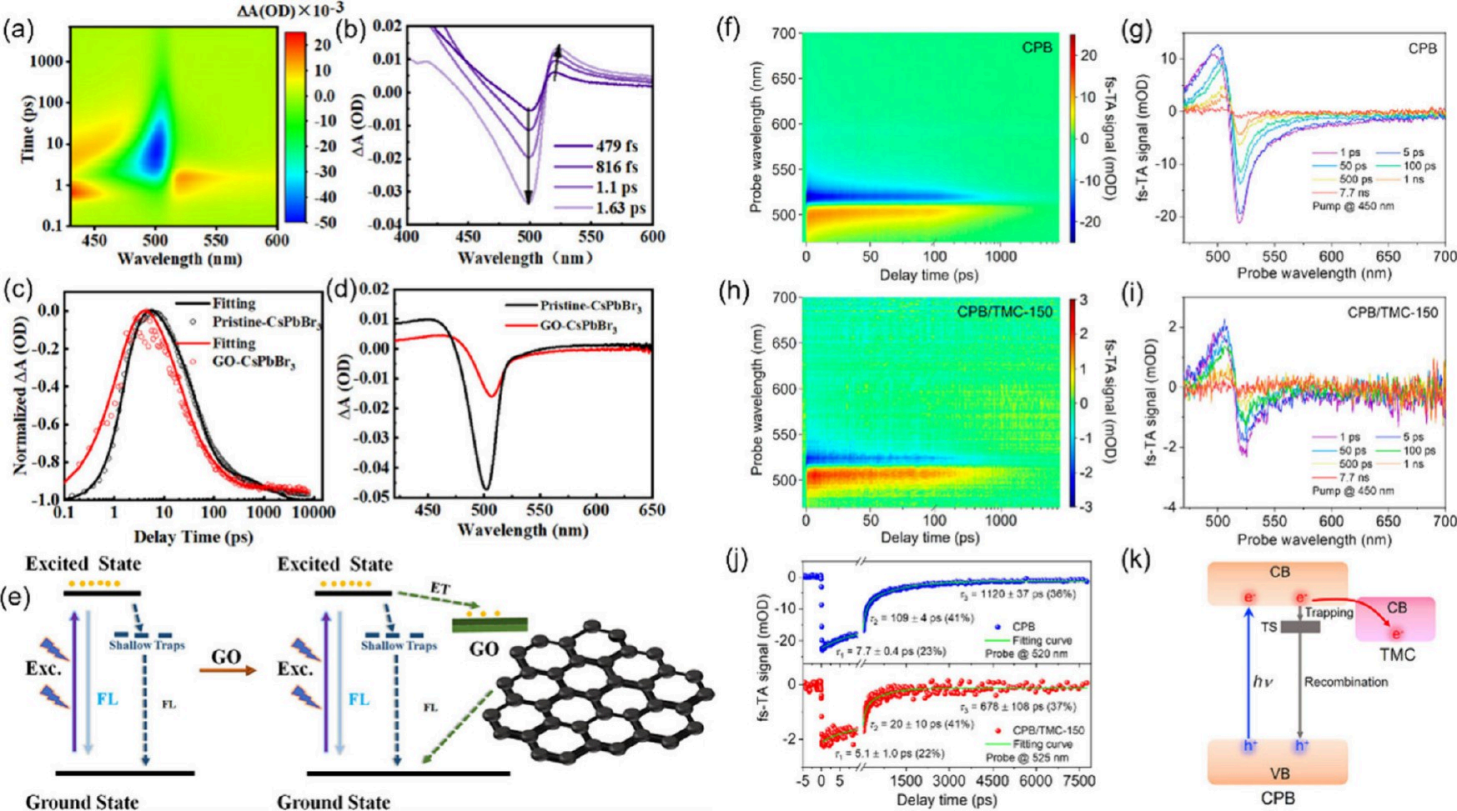
(a) Two-dimensional map of fs-TAS spectra of CsPbBr_3_ and (b) corresponding TAS spectra at diverse delay times. (c) Recovery kinetics of hot charge carrier absorption at 500 nm and (d) TAS spectra at 10 ps of CsPbBr_3_ and CsPbBr_3_/GO. (e) Schematic illustration of charge excitation and transfer process in CsPbBr_3_ and CsPbBr_3_/GO. Reproduced with permission from ref (175). Copyright 2023, American Chemical Society. fs-TAS spectra counter-maps of (f) Cs_4_PbBr_6_ and (h) Cs_4_PbBr_6_/TiO_2_. TAS spectra at different delay times recorded on (g) Cs_4_PbBr_6_ and (i) Cs_4_PbBr_6_/TiO_2_. (j) TAS decay traces of Cs_4_PbBr_6_ and Cs_4_PbBr_6_/TiO_2_. (k) Illustration of the exciton evolution process in Cs_4_PbBr_6_/TiO_2_. Reproduced with permission from ref (176). Copyright 2023, American Chemical Society.

The variation of charge carrier dynamics resulting from the formation of heterostructure can be tracked real-time by analyzing the TAS spectrum. For a typical example, the charge separation and transfer mechanism in a CsPbBr_3_/GO heterostructure was investigated in detail using TAS spectroscopy^175^ (Figure 14a–d). After GO attachment, the SE and GSB intensities show an evident decline in CsPbBr_3_/GO in comparison to pristine CsPbBr_3_. The triple exponential function fitted kinetics further illustrate that CsPbBr_3_/GO exhibits a shorter carrier lifetime (τ_1_ = 1.29 ps, τ_2_ = 18.6 ps, τ_3_ = 105 ps) than that of blank CsPbBr_3_ (τ_1_ = 1.35 ps, τ_2_ = 38.37 ps, τ_3_ = 1460 ps). The duration τ_1_ can be interpreted as the relaxation period for hot carriers within the intraband, while τ_2_ signifies the transfer process where electrons migrate toward GO. τ_3_ denotes the instance where carriers become entrapped in shallow defect states. The faster decay behavior suggests effective electron transfer from CsPbBr_3_ to GO with reduced trapping by shallow defect states in CsPbBr_3_. As shown in Figure 14e, the underlying mechanisms could be explained as the adherent of CsPbBr_3_ on GO induces robust chemical interaction at the interface. GO containing oxygen functional groups could be deemed as a semiconductor, with sp^2^ carbon functioning as the CB and sp^3^ carbon serving as the VB, hence providing a strong driving force for the electron transfer.

Another case is to verify the directional exciton diffusion in the Cs_4_PbBr_6_/TiO_2_ mesocrystal (CPB/TMC) heterostructure.^176^ With a lower intensity of the TAS signal, CPB/TMC also has a significantly shortened charge carrier lifetime compared to that of pristine Cs_4_PbBr_6_ (Figure 14f–k). Since τ_1_ and τ_2_ are assigned to intrinsic defects trapping, it demonstrates fast photoinduced electron transfer from Cs_4_PbBr_6_ to TiO_2_. Specifically, the calculated electron transfer rate constants based on the triexponential fitting results show that the first component *k*_1_ = 6.6 × 10^10^ s^–1^ is almost three times higher than the reported conventional cesium lead halide/TiO_2_ systems, demonstrating the accelerated charge transfer through a directional diffusion from the plane center to the edge region of TiO_2_.

## 5 Surface Reactions of HP/2D Material Heterostructure Photocatalysts

Constructing an HP/2D material heterostructure has been demonstrated as a superior strategy for achieving higher photocatalytic activity since numerous HP/2D material heterostructures achieved commendable performance in diverse photocatalytic applications including H_2_ evolution, CO_2_ reduction, organic synthesis, and pollutants degradation. Undoubtedly, the exceptional photocatalytic performance of HP/2D material heterostructures is closely correlated with their intriguing surface properties which promote the surface reactions.

### 5.1 Catalytic Activity

The activity of photocatalysts is markedly influenced by the charge transfer. Integrating HP with 2D materials (e.g., graphene, MXenes, and TMDs) that possess high conductivity can significantly facilitate electron transfer from the photocatalyst to the reaction medium. It is reported that some TMDs are ideal replacements for noble metals and promise to outperform them in terms of functionality. Besides, their excellent stability enables they will be resistant to strongly acidic environments such as HX (X = Cl, Br, I) aqueous solutions where moisture-sensitive HPs can maintain stability. Consequently, superior H_2_ evolution performances are observed in HP/TMD heterostructures. For example, MoS_2_ QDs show a much higher electrocatalytic HER potential compared to Cs_3_Bi_2_I_9_, suggesting a remarkable HER activity. After loading MoS_2_ QDs on Cs_3_Bi_2_I_9_, the obtained MoS_2_ QDs/Cs_3_Bi_2_I_9_ exhibit significantly smaller EIS radius and higher photocurrent density in comparison to pristine Cs_3_Bi_2_I_9_ and MoS_2_ QDs, respectively, demonstrating lower charge transfer resistance from MoS_2_ QDs/Cs_3_Bi_2_I_9_ to the liquid environment. As a result, the optimal MoS_2_ QDs/Cs_3_Bi_2_I_9_ sample attained a H_2_ evolution rate of 6.09 mmol g^–1^ h^–1^, which is 2.4 and 8.8 times higher than those of Pt-loaded Cs_3_Bi_2_I_9_ (Pt/Cs_3_Bi_2_I_9_) and bare Cs_3_Bi_2_I_9_, respectively.^91^ Similar results are also obtained by MAPbI_3_/MoS_2_, MoSe_2_/MAPbBr_3-*x*_I_*x*_, and MAPbI_3_–Ti_3_C_2_T_*x*_ with enhanced photocatalytic H_2_ evolution performance. Another representative example is the black phosphorus/MAPbI_3_ (BP/MAPbI_3_) heterostructure, which possesses more favorable electrocatalytic HER activity as it displays a lower onset potential (642 mV) than that of pure MAPbI_3_ (780 mV) in the *I–V* curves. Additionally, the EIS Nyquist plots evidently illustrate that BP/MAPbI_3_ has a smaller semicircular diameter than that of MAPbI_3_, demonstrating accelerated interfacial charge transfer with the aid of high-conductive BP few-layer. Accordingly, an impressive photocatalytic H_2_ evolution rate up to 3742 μmol g^–1^ h^–1^ was achieved by BP/MAPbI_3_ with 1.2% BP loading amount, which is 18 times over that of Pt/MAPbI_3_ (192 μmol g^–1^ h^–1^).^82^

### 5.2 Catalytic Selectivity

**Figure 15:**
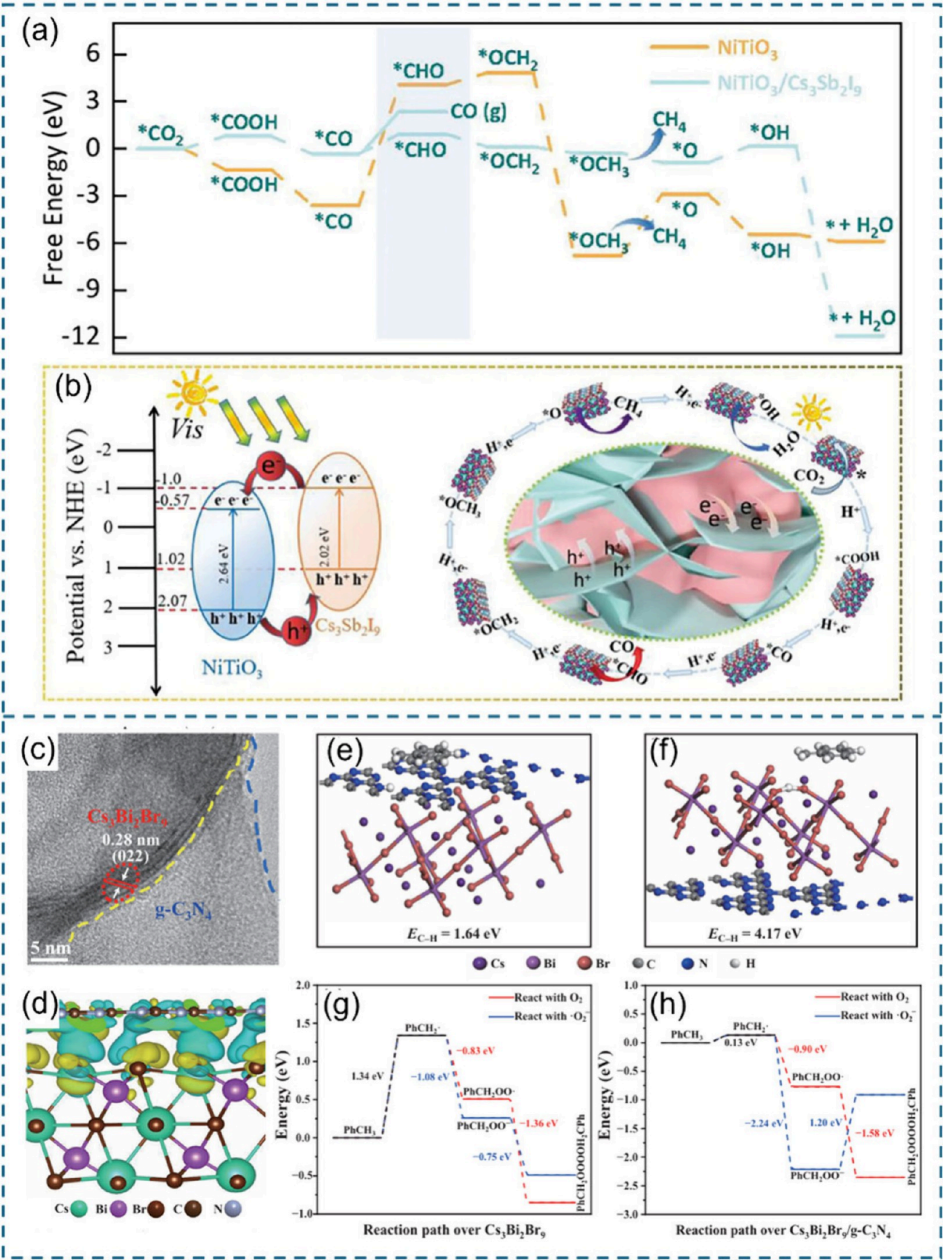
(a) Energy barrier variation of the transformation of CO_2_ to CH_4_ and CO over NiTiO_3_ and NiTiO_3_/Cs_3_Sb_2_I_9_. (b) Mechanism of photocatalytic reduction of CO_2_ to CH_4_ by NiTiO_3_/Cs_3_Sb_2_I_9_. Reproduced with permission from ref (178). Copyright 2024, Wiley-VCH. (c) TEM image of the heterointerface between Cs_3_Bi_2_Br_9_ and g-C_3_N_4_. (d) Charge density difference of Cs_3_Bi_2_Br_9_/g-C_3_N_4_. Breaking of toluene C–H bond on (e) Cs_3_Bi_2_Br_9_ and (f) g-C_3_N_4_ surfaces of Cs_3_Bi_2_Br_9_/g-C_3_N_4_. Energy diagram of the toluene oxidation pathway over (g) Cs_3_Bi_2_Br_9_ and (h) Cs_3_Bi_2_Br_9_/g-C_3_N_4_. Reproduced with permission from ref (141). Copyright 2022, Springer.

Photocatalysis reactions such as CO_2_ reduction and organic compound conversion entail intricate reaction steps and the formation of intermediates as well as byproducts. Thus, achieving high selectivity for target products is extremely important. Constructing HP/2D material heterostructure can effectively modify the properties of photocatalysts from aspects of physical, chemical, and band structures, contributing to high photocatalytic activity and selectivity. Taking CO_2_ reduction as an example, the process of CO_2_ conversion requires electron and proton transfers. However, as water is a common supplier of proton, a more thermodynamic favorable reduction of proton to H_2_ will occur and severely compete with CO_2_ reduction.^177^ Therefore, a hydrophobic surface is beneficial to suppressing the unwanted H_2_ production induced by H_2_O adsorption. For instance, NiTiO_3_ nanoflowers composed of NiTiO_3_ nanosheets were used as support for the in situ growth of Cs_3_Sb_2_I_9_. The obtained NiTiO_3_/Cs_3_Sb_2_I_9_ exhibits a higher hydrophobic surface compared to a single NiTiO_3_ since a larger contact angle is demonstrated. And this is conducive to reducing the competition of the HER. Subsequently, the photocatalytic CO_2_ reduction process was monitored by in situ diffuse reflectance Fourier transform infrared spectroscopy (DRIFTS). It shows that during the photocatalytic reaction using NiTiO_3_/Cs_3_Sb_2_I_9_, the intensity of characteristic peaks belonging to *COOH, *CO, *OCH_2_, and *OCH_3_ which are the key intermediates for the conversion from CO_2_ to CH_4_ increase gradually with prolong of illumination. Based on this, the potential reaction pathways are further proposed by DFT calculation. For NiTiO_3_/Cs_3_Sb_2_I_9_, the high energy demand for *CO to convert to CO contributes to the suppression of CO desorption. Moreover, the energy consumption of *CHO generation from *CO for NiTiO_3_/Cs_3_Sb_2_I_9_ (Δ*G* = 1.26 eV) is significantly lower than that for NiTiO_3_ (Δ*G* = 7.68 eV), which paves a favorable way to CH_4_ formation (Figure 15a). As a result, given the intimate interface, well-matched band structures, and intriguing surface properties, NiTiO_3_/Cs_3_Sb_2_I_9_ achieves an impressive CH_4_ yield with a high selectivity of 88.6% (Figure 15b).^178^

Conversion of the C–H bond of a hydrocarbon (such as toluene) to value-added chemicals through photocatalysis proposes promising insights into organic synthesis. However, the strong benzylic C–H bond with chemical inertia makes its dissociation a rate-limiting step in the selective oxidation process. A 2D/2D Cs_3_Bi_2_Br_9_/g-C_3_N_4_ heterostructure was successfully constructed for highly selective C–H bond oxidation (Figure 15c).^141^ On the one hand, the formed type II heterojunction drives the separation and transfer of photoexcited electrons and holes, which promote the generation of active species (e.g., ^•^O_2_^–^ and h^+^) and intermediates. On the other hand, the incorporation of g-C_3_N_4_ modifies the surface energy of Cs_3_Bi_2_Br_9_ and alters the reaction pathway, resulting in enhanced selective production of benzaldehyde. DFT calculations reveal that in the Cs_3_Bi_2_Br_9_/g-C_3_N_4_ heterostructure, the toluene molecule is inclined to adsorb on the surface of g-C_3_N_4_ since the toluene adsorption energy (*E*_ads_) on the surface of g-C_3_N_4_ is larger than that of the Cs_3_Bi_2_Br_9_ surface. In particular, the C–H bond dissociation energy (*E*_C–H_) on the surface of g-C_3_N_4_ (1.64 eV) is significantly lower than that on the Cs_3_Bi_2_Br_9_ surface, meaning that the activation of the C–H bond preferable to occur on the g-C_3_N_4_ surface. When a hole is introduced into the system, the ^E^_C–H_ is reduced to 0.13 eV, further proving that holes on the VB of g-C_3_N_4_ play a significant role in the C–H bond cleavage. Moreover, the toluene oxidation processes on pristine Cs_3_Bi_2_Br_9_ and Cs_3_Bi_2_Br_9_/g-C_3_N_4_ are calculated and compared in Figure 15e-h. Obviously, the existence of g-C_3_N_4_ provides a more feasible path for the generation of benzaldehyde over Cs_3_Bi_2_Br_9_/g-C_3_N_4_ and consequently contributes to a remarkable benzaldehyde selectivity of 90%. In contrast, due to the formation of benzyl alcohol, the blank Cs_3_Bi_2_Br_9_ shows only 65% selectivity to benzaldehyde.

### 5.3 Catalytic Stability

Stability is a crucial issue in HP-based photocatalysis applications. The intrinsic ionic characteristic renders HP unstable under ambient conditions. Various factors at the interfaces between HP and the surrounding environment, including heat, light, and moisture, can cause the irreversible decomposition of HP and significantly hinder its photocatalytic activity. Integrating HP with 2D materials that have excellent physicochemical stability gives rise to enhanced photocatalytic, improved durability, and increased efficiency in solar-driven processes.

**Figure 16:**
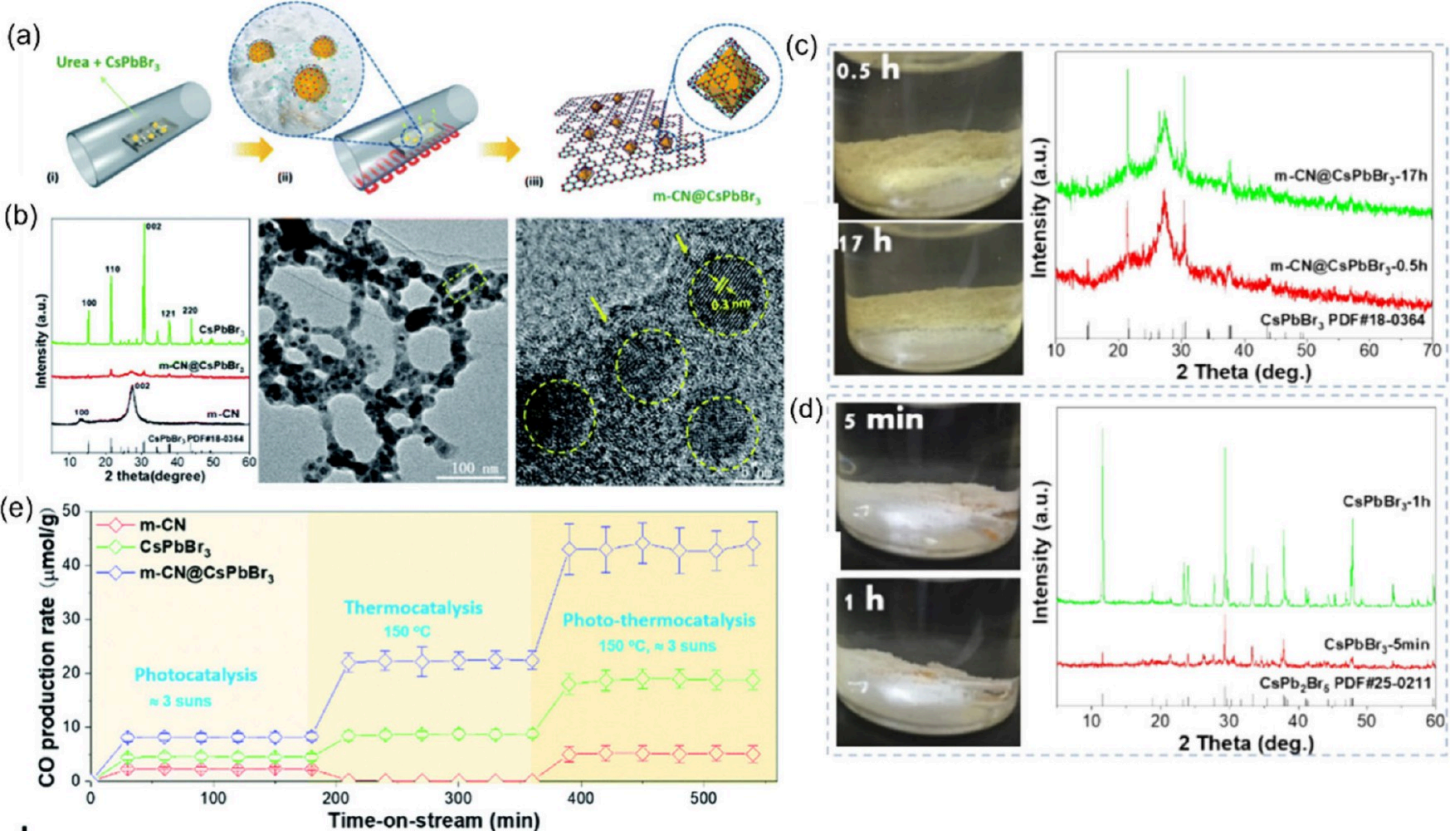
(a) Illustration of the fabrication process of m-CN@CsPbBr_3_. (b) XRD patterns and TEM images of m-CN@CsPbBr_3_. Photographs and corresponding XRD patterns of (c) m-CN@CsPbBr_3_ and (d) pristine CsPbBr_3_ placed in water at different times. (e) CO_2_ reduction performance under different conditions. Reproduced with permission from ref (110). Copyright 2022, The Royal Society of Chemistry.

A special 2D g-C_3_N_4_-coated CsPbBr_3_ (m-CN@CsPbBr_3_) composite was successfully prepared via a molten-salt method using urea and CsPbBr_3_ as the precursor.^110^ Under high temperature in a N_2_ atmosphere, CsPbBr_3_ is converted into a solution state and then encapsulated by mesoporous g-C_3_N_4_ with the cooling process (Figure 16a,b). Thermogravimetric analysis (TGA) demonstrates satisfactory stability of m-CN@CsPbBr_3_, as no decomposition is observed below 200 °C. Notably, m-CN@CsPbBr_3_ exhibits superior water stability since it can maintain its original morphology and crystal phase after immersion in water for 17 h. In contrast, pristine CsPbBr_3_ is suddenly degraded after soaking in water for only 1 h, with an obvious phase transition (Figure 16c,d). The protection from m-CN not only improves the stability but also promotes the CO_2_ catalytic reduction performance. As illustrated in Figure 16e, m-CN@CsPbBr_3_ outperforms bare m-CN and CsPbBr_3_ under the conditions of light, heat, and light with heat, exhibiting remarkable photothermocatalytic CO production of 42.8 mmol g^–1^ h^–1^.

Many studies have reported that HPs can maintain stability in nonpolar organic solvents and exhibit satisfactory performance on dye degradation. However, pollutant degradation in an aqueous environment is still a challenge for HPs. Recently, some HP/2D materials heterostructure (e.g., CsPbBrCl_2_/g-C_3_N_4_, Bi_2_WO_6_/Cs_2_AgBiBr_6_, CsPbBr_3_–MoS_2_-GO) have demonstrated their capability to degrade organic pollutants in water environment. For example, by utilizing an amine functional group (−NH_2_ and NH) and a terminal cyano group (−CN) on the surface of g-C_3_N_4_ which leads to a positive charge, incorporating CsPbBrCl_2_ with g-C_3_N_4_ could improve the surface zeta potential, which facilitates better adsorption of anionic dyes. The obtained CsPbBrCl_2_/g-C_3_N_4_ possesses impressive stability and strong affinity for Eosin B. Combining with the efficient charge transfer and separation boosted by the type II heterostructure, CsPbBrCl_2_/g-C_3_N_4_ exhibits remarkable photocatalytic performance by removing 94% Eosin B in 120 min and shows negligible decrease after three cycles of use. Another study proved that nitrogen-doped graphene quantum dots (N-GQDs) can enhance the stability of CsPbIBr_2_. The interactions between −COO and Pb^2+^, as well as between -NH and halide ions (Br^–^ and I^–^), contribute to a strong combination of N-GQDs and CsPbBrI_2_. As a result, the surface of CsPbBrI_2_ is uniformly covered by N-GQDs, which demonstrates pronounced stability in water.^179^

The excellent photocatalytic performance of the HP/2D material heterostructures could also be attributed to their enhanced thermostability and photostability. Continuous exposure under light irradiation will inevitably induce the decomposition and phase segregation of HPs and impair their photocatalytic activity. Introducing 2D materials (e.g., GO, TMDs, h-BN) with high thermal and electric conductivity can effectively inhibit the accumulation of heat and light-generated charge carriers in HPs through heat and charge extraction.^37^ This stability enhancement can also be achieved by the construction of a type II or Z-scheme heterojunction with efficient charge transfer and separation.

## 6 Conclusions and Perspectives

Herein, HP/2D material heterostructure photocatalysts have been systematically reviewed, which is timely. First, we briefly introduced the intrinsic properties of HPs and 2D materials and proposed the advantages of their combination. 2D materials that possess tunable morphology and electronic structure as well as fast charge transfer mobility serve as ideal companions for HPs. The formation of an HP/2D material heterostructure unleashes the considerable potential of both HP and 2D materials, facilitating unprecedented photocatalytic activity. Then, the construction of HP/2D material interfaces is thoroughly investigated by discussing preparation strategies and architecture modes of interfaces. Typical HP/2D material heterostructure photocatalysts in which 2D materials act as cocatalysts or semiconductors are comprehensively summarized and compared. The abundant variety and unique attributes of 2D materials enable the formed heterostructures to be versatile in function, showcasing excellent photocatalytic performance in diverse applications. Furthermore, the charge carrier behaviors at the HP/2D material interface, which could significantly affect the photocatalysis performance, are vividly elaborated and combined with examples of advanced time- and space-resolved techniques. Finally, the enhanced surface photocatalytic reactions induced by favored surface properties of the HP/2D material heterostructure are analyzed. Despite the impressive progress achieved by emerging HP/2D material heterostructures in the field of photocatalysis, they are still in their infancy and are far from practical applications. Continuous endeavors are required to reveal the underlying mechanisms and obtain higher overall performance of HP/2D material heterostructure photocatalysts. Hence, we propose several challenges and directions for HP/2D material heterostructure photocatalysts to provide insightful guidance for ongoing research and development.

### 1) Constructing an Intimate Interface

The interface plays a pivotal role in the HP/2D material heterostructure, which could directly determine its photocatalysis performance. However, building an intimate interface between the HP and 2D materials is still challenging. The soft structure owing to the intrinsic ionic composition of HP is distinct from the hard structure of metal oxides or the covalent-bonded 2D materials (e.g., TMDs, g-C_3_N_4_), which makes the in situ growth of one of them on the other through an epitaxial extension way difficult. Epitaxial growth generally requires a close lattice spacing of selective facets of both materials to ensure lattice matching. Moreover, having common elements between two materials is also favorable for epitaxial growth. Therefore, in most cases, achieving strong interaction between HPs (e.g., CsPbBr_3_, MAPbI_3_) and 2D materials (MoS_2_, g-C_3_N_4_, Ti_3_C_2_) with different bonding, crystal lattice, and element composition is difficult.^180,181^ To overcome this problem, some possible approaches are proposed. (i) Regulation of facets and lattice spacings. Through optimization of exposure facets and crystallization of HPs by tailoring the growth temperature and solution concentration, the corresponding different lattice spacings are likely to provide more opportunities for the interface combination with 2D materials. Alternatively, high quality HP crystal can be achieved through facilely spin coating the HP liquid precursors on the substrate followed by slow evaporation under ambient conditions.^182^ Additionally, halogen regulation is a feasible way to continuously alter the lattice spacings of HPs and is conducive to lattice matching. (ii) Introduction of bridging mediates at the interface. Some ions, molecules, and ligands may be able to coordinate with surface atoms, and bridges connecting the surfaces of two materials are expected to form. Furthermore, intercalating intermediates that have the common elements of HP and 2D materials such as CsPbBr_3_–PbSBr-MoS_2_ or CsPbBr_3_–PbSeBr-MoSe_2_ are hopeful to achieve epitaxial extension, but the proper introduction of these mediates during the preparation process still needs to be investigated. (iii) elemental doping. Elemental doping is a facile way to modify the surface coordination of the materials. For example, doping metal ions in 2D materials to create the same hexacoordinated surface as the B site ions of HPs might provide symmetric coordination environments and facilitate the interfacial connection. (iv) Vapor phase epitaxy. This is a van der Waals heteroepitaxy strategy without the involvement of liquid and can be conducted through thermal evaporation and chemical vapor deposition (CVD). Specifically, HP precursors will grow on 2D materials substrates put downstream of the tube during a heating protocol with flowing of argon gas. The crystallinity, exposure facet, and thickness of grown HP can be precisely controlled via adjusting the pressure, temperature, time, and gas flowing rate of the reaction system.^183^ This strategy has shown huge potential for the fabrication of high-quality HP/2D material van der Waals heterostructures. It should be noted that many stable HP/2D material interfaces can also be obtained by a self-assembly method using electrostatic force, and their impressive effects on photocatalysis have been widely demonstrated. Hence, it is difficult to conclude which approach is preferable for constructing a desirable HP/2D material interface. More efforts from aspects of the experiment, characterization, and theoretical calculation are needed in this issue.

### 2) Improving the Stability and Photocatalytic Activity

Stability is a persistent issue of photocatalysts, especially for HPs with low water resistance. Though considerable progress has been made in the stability of HPs through various strategies such as composition regulation, encapsulation, and ligand-assistant protection, the stability of HPs is still far from satisfactory compared to traditional semiconductor photocatalysts. One of the most significant questions are the instability of HPs in aqueous environments. Up until now, the use of HPs for photocatalytic water splitting has yet to be realized. Moreover, the majority of studies on HP degradation of pollutants are conducted in organic solvents, while their stability and performance in water solutions are still unsatisfactory. When integrating HPs and 2D materials, how to simultaneously unleash both of their advantages is essential. An ideal HP/2D material heterostructure should exhibit extensive light absorption capability, highly efficient charge separation and transfer, abundant surface active sites, and excellent stability. The high flexibility in modifying the structure/dimension of HPs and 2D materials opens up possibilities for developing durable heterostructures. It is proposed that core–shell configuration is a promising structure that could simultaneously achieve high photocatalytic activity and stability, where light attractive HP is encapsulated by 2D material with a large surface area and rich reactive sites. However, one should devote consideration to material selection and structure regulation. For example, the thickness of the 2D material which covers HP should be appropriate. A thicker encapsulation layer can effectively prevent HP from degradation by water; however, it also reduces the light transmittance, thereby restricting the HP’s light utilization. Equally importantly, modifications can further enhance the photocatalytic performance via tuning the separation and transfer pathway of photogenerated charge carriers. It is reported that incorporating Au as a mediator in a core–shell structure can change the charge transfer pathway from type II of CsPbBr_3_/TiO_2_ into a direct Z-scheme of CsPbBr_3_/Au/TiO_2_, resulting in stronger photocatalytic redox potential.^60^ Ligand engineering can also improve the stability of the HPs. The strong binding effect between ligands and perovskite surfaces prevents HP from decomposition in aqueous environment. It is reported that novel multi-amine ligand, *N*′-(2-aminoethyl)-*N*′-hexadecylethane-1,2-diamine (AHDA) could create strong chelating action at the surface of CsPbI_3_ nanocrystals via a hot injection method. Protonated AHDA exhibits a strong binding affinity to the PNC surface lattice, with a greater binding than conventional oleylammonium ligands. The resulting chelating interaction significantly impedes the dynamic desorption of surface ligands, thereby facilitating the stabilization of CsPbI_3_ PNCs against a wide range of environmental stimuli.^184^ Consequently, the rational engineering of the composition and structure holds great promise for developing HP/2D material heterostructures that exhibit exceptional stability and activity.

### 3) Expanding the Members of HP/2D Material Heterostructures and Photocatalytic Applications

Despite numerous HP/2D material heterostructure photocatalysts that have been reported, there still remains large room for the exploration of emerging HPs and 2D materials that are more compatible with each other and could synergistically enhance the photocatalysis performance. In terms of lattice structure, most HPs in documented HP/2D material heterostructures are 3D HPs such as CsPbBr_3_ and MAPbI_3_. However, combinations of 2D and quasi-2D HPs, particularly Ruddlesden–Popper (RP) HPs and Dion–Jacobson (DJ) HPs, with 2D materials are seldom reported. Compared to traditional 3D HPs, 2D or quasi-2D HPs are exempt from the limitations of tolerance factor and have substantial freedom in functional organic cation selection, which enables them to have tunable photoelectronic properties and satisfactory stability. Moreover, the large organic cations, which connect the perovskite layers through van der Waals force or hydrogen bonding, potentially interact with 2D materials to form stable heterointerfaces. Thus, developing 2D HP (with respect to lattice structure)/2D material heterostructures could be a promising research direction. On the other hand, 2D organic polymer semiconductors such as 2D COFs could be a new ideal partner for HPs. The large surface area, abundant active sites, and functional groups, as well as the special porous structure, make 2D COFs excellent substrates for HPs to construct heterostructures. More importantly, recent studies have demonstrated that COFs and HPs are beneficial for each other during their crystalline processes.^185,186^ The metal sites of HP could interact with ligands or functional groups of 2D COFs, leading to uniform and ordered crystal phases; as a result, the charge transfer, conductivity, and stability of materials are significantly enhanced. In comparison with traditional 2D metal oxides, the enriched surface functional groups of 2D COFs are also conducive to improving the selectivity in catalysis reactions. Of equal significance, the escalating energy crisis and environmental pollution necessitate the adoption of photocatalytic reactions that exhibit heightened efficiency, such as the cooperative coupling of organic synthesis with energy fuel production. In this type of reaction, the production of H_2_ or the reduction of CO_2_ can be seamlessly integrated with the activation of X–H bonds (where X = C, N, O, S).^187^ Such a system offers a transformative approach where organic synthesis replaces the consumption of sacrificial reagents. This not only expedites the sluggish oxidation half-reaction but also achieves the simultaneous production of renewable clean energy and value-added chemicals.^188^ Likewise, the concurrent photoredox co-upcycling of CO_2_ and plastic waste or biomass into syngas and value-added feedstocks, respectively, holds promise for addressing pressing energy and environmental challenges.^189−191^ It is undeniable that the development of novel HP/2D heterostructure photocatalysts will continue to advance, leading to substantial improvements in solar-driven applications.

### 4) Revealing Interfacial Mechanisms through a Rational Combination of Characterizations and Theoretical Calculations

The interfacial processes are very intricate and involve interface interaction, charge transfer and separation, and various surface reactions. To elucidate these complex interfacial issues, characterizations and theoretical calculations have been commonly conducted in studies in recent years. However, caution and consideration are necessary in their practice. The structure model and simulated reaction environment of theoretical calculations may greatly differ from the actual conditions. Thus, the correct parameters based on experimental characterizations are crucial. To this end, in situ techniques (e.g., TRPL, TAS, KPFM, time-resolved TEM, in situ Raman spectroscopy) with real-time monitoring capabilities are encouraged to observe the dynamic interfacial processes under operational conditions. Coupling these experimental approaches with high-fidelity theoretical calculations will provide deeper insights into the underlying mechanisms. Moreover, theoretical calculations and machine learning^192^ should play a prediction role that guides the materials synthesis and reaction processing rather than merely verify the experiment results.

### 5) Advancing Photocatalysis to Electrocatalytic and Photoelectrochemical Systems

While photocatalysis offers the advantage of operating under mild conditions and utilizing solar energy directly, its efficiency is often limited by factors such as charge recombination and material stability. The incorporation of HPs and 2D material heterostructures into electrocatalytic and photoelectrochemical (PEC) systems presents a promising direction for enhancing energy conversion technologies. In electrocatalytic applications, HP/2D heterostructures can function as effective catalysts for reactions such as the hydrogen evolution reaction (HER) and oxygen evolution reaction (OER). The distinctive properties of 2D materials, including their high surface area and tunable electronic structures, combined with the light-absorbing capabilities of HPs, enable enhanced catalytic activity and stability. More efficient energy conversion processes can be expected due to synergistic effects at the interface. On the other hand, PEC systems aim to combine the benefits of both approaches by utilizing solar energy to drive electrochemical reactions, potentially offering higher efficiencies than traditional photocatalysis while minimizing the necessity for external electricity. In PEC systems, HP/2D heterostructures can serve as photoanodes or photocathodes, thereby facilitating a cleaner and more sustainable solar-driven energy conversion process, for instance, the transition from “gray hydrogen” to “green hydrogen”. By leveraging the unique properties of HPs and 2D materials, researchers can pave the way for the development of more efficient and environmentally friendly energy conversion processes, contributing to the advancement of clean energy technologies.

To sum up, HP/2D material heterostructure photocatalysts have demonstrated their great potential for solar energy harvesting. Practical applications of HP/2D material heterostructure photocatalysts can be expected to expand with continuous efforts. Also, HP/2D material heterostructures are hoped to be ideal platforms for studying photocatalytic mechanisms, providing insights for future study.
